# Malaysian older persons’ perceptions about falls and their desired educational website characteristics: A qualitative study

**DOI:** 10.1371/journal.pone.0270741

**Published:** 2022-07-07

**Authors:** Cheah Ping Ng, Devinder Kaur Ajit Singh, Maw Pin Tan, Saravana Kumar

**Affiliations:** 1 Physiotherapy Programme & Centre for Healthy Ageing and Wellness, Faculty of Health Sciences, Universiti Kebangsaan Malaysia, Kuala Lumpur, Malaysia; 2 Division of Geriatric Medicine, Department of Medicine, Faculty of Medicine, University of Malaya, Kuala Lumpur, Malaysia; 3 UniSA Allied Health and Human Performance, City East Campus, University of South Australia, Adelaide, South Australia, Australia; Indian Institute of Management Bangalore (IIM-B), INDIA

## Abstract

Falls is a common and debilitating condition among the older population, intensifying the need to educate older persons about falls. Technology advancement enables effective and efficient delivery of falls education to the older population. However, there is paucity of information on the perception of Malaysian older population on falls and their preferred website characteristics such as font size, design, layout, colour, navigation, and use of graphics or videos. Physiological changes in vision, cognition and psychomotor skills can affect how the older persons use the website. As Malaysia is a multicultural country, the needs of the website characteristics and falls perception of older persons may differ greatly. The aim of this study was to explore the perceptions of the older persons about falls and their desired website characteristics. Twenty-five community-living older persons (n = 25) of age 60 years and above were involved in the focus group discussions. NvivoTM software was used for data management and thematic analysis was undertaken. Emerging themes included ’Perceptions of falls in older persons’, ’Actions taken when falls occurred’, ’Perceived prevention strategies for falls’ and ’End user requirements for falls educational website’. Falls were perceived as both an avoidable and a non-avoidable incident. Although the participants mentioned physical activity and home hazard modifications as strategies to prevent falls, they mainly discussed self-initiated precautionary approaches in falls prevention. Regarding desired website characteristics, the participants emphasized on easily readable text, appealing design, clear information, use of images/videos, and simple website navigation. Special requirements for colour selection and multi-language options were also raised. The delivery of falls education through website can be made possible by understanding the perception of older persons about falls and their requirements for the website. This is especially important as ethnic and cultural influences may play a role on their perceptions about falls and desired website characteristics.

## Introduction

The number of older persons worldwide is increasing rapidly [1]. Similarly, the number of older persons in Malaysia is growing with an expectation of reaching the ageing society in year 2030, whereby older persons will constitute 15% of its population [2]. Parallel to this, there will be a rise in age-related negative consequences including falls among older persons. The incidence of a fall and recurrent falls was about 8.5 and 3.2 per 100 person-years respectively among Malaysian older persons [3]. Higher prevalence is reported among older persons residing in the institutions and with poor nutritional status [4]. Falls in older person can cause injuries, admissions to hospital, increased dependency in activities of daily living, deaths and re-admissions to hospital for recurrent falls [5–7]. In addition, it increased economic burden of medical costs and the needs of social care [8, 9].

Evidence-based falls prevention strategies have been recommended in available guidelines. However, various factors influence its implications and outcomes. Older persons’ awareness and knowledge about falls are vital components for their engagement in falls prevention strategies [10, 11]. Nonetheless, low self-perceived risk of falls, falls viewed to be unimportant, denotations of the label ’old’ or ’frail’, and a view of falls prevention strategies as personally irrelevant or unnecessary were common among older persons [12–17]. Also, older persons often viewed falls as something that occurred due to ageing, which is unavoidable [13, 15, 17, 18]. These suggest that there is a need to provide continuous awareness and education regarding falls prevention to older persons. In the technological era, it is possible to educate, engage and empower older persons utilizing online platforms, such as websites. In lieu to the educational effort, it demonstrates that the usage of websites to assist in education on falls prevention and intervention is almost minimal to none in Malaysia and the region of Southeast Asia [19].

However, older persons’ access to websites and other online resources is not without barriers. Age related changes in vision, hearing, fine movement control, memory and attention could cause usability problems for older persons [20]. These changes need to be considered when developing websites specifically for older persons. Previous studies that evaluated the use of websites among older persons, drew attention to the importance of developing a senior friendly website [21, 22]. This is becoming more crucial, especially during the current COVID-19 pandemic, whereby physical access to health care is restricted and more people are engaging with online resources. Regardless, most health-related websites for older persons were found to be not optimally designed to promote its use among older population [23].

There are a number of guidelines on the development of websites for older adults [24–26]. However, the guidelines are presented by the authors or organizations differently. For example, in Zaphiris et al. [26], 38 final guidelines are grouped into eleven distinct categories, such as target design, use of graphics, navigation, browser window features, content layout design, links, user cognitive design, use of colour and background, text design, search engine, user feedback and support. For Web Content Accessibility Guidelines (WCAG) 2.0, 20 points are categorized under four headings which are perceivable information and user interface, operable user interface and navigation, understandable information and user interface, and robust content and reliable interpretation [25]. As for the National Institute on Aging, they divided 25 points into four categories, namely designing readable text for adults, presenting information to older adults, incorporating other media, and increasing the ease of navigation [24]. Although guidelines on developing a senior friendly website are available [24–26], most websites failed to comply with these guidelines [21, 27]. In addition, the needs and requirements of older persons as the end-users, were not taken into consideration when designing websites for their use [22, 28, 29]. In the study by Hart et al. [21], the significance of using both guidelines and usability testing when designing websites for older persons was highlighted. A recent review suggested that older persons preferred website characteristics such as easily readable text, simple design, consistent layout and easy to use web navigations [30]. One of the limitation identified in this review was that much of the literature on this topic was confined to Western countries. Too little is known about older persons’ preferences for website characteristics in Malaysian local settings that constitute of a multiethnic society. Prior to the development of a senior friendly website to deliver falls awareness and education to older persons, it is important to understand their perspectives of falls and what are their desired website characteristics. Therefore, this qualitative study explored older persons’ perceptions about falls and their desired educational website characteristics.

## Methods

This study was approved by the Secretariat for Research and Ethics of Universiti Kebangsaan Malaysia (Reference: NN-2018-065). Written consent was given by every participant after reading the information sheet. Participants were informed in both verbal and written format, that participation in this study is completely voluntary and they may withdraw from the study at any time without any consequences.

### Design

This qualitative study used focus group discussions method to explore older persons’ perceptions about falls and their desired website characteristics. A group of older persons meeting the inclusion criteria were invited to discuss on a predetermined topic and the entire session was guided by the researcher and an assistant. Focus group discussions method was selected as falls is an issue of common interest among the older persons and group dynamics can facilitate optimal idea sharing. Reporting of this study was guided by the COREQ (COnsolidated criteria for REporting Qualitative research) checklist [31].

### Participants and setting

Participants were recruited using purposive sampling method [32]. This form of sampling was employed to capture the comprehensive views of Malaysian older persons from different ethnicity, age groups, gender, and computer literacy levels. Community dwelling older persons aged 60 years and above, able to speak and understand English language were recruited from two senior citizens’ clubs located in Petaling Jaya, Selangor. Those with depression, dementia, and uncorrected hearing or vision impairment were excluded for the study. Depression status was assessed using the Geriatric Depression Scale (cut-off value ≥5), while cognitive status was assessed using the Visual Cognitive Assessment Test (cut-off value <18). Sample size is considered adequate when the researcher is assured that the topic was discussed in sufficient detail and no new information emerged with the on-going focus group [33].

### Data collection

A total of 45 participants were screened for eligibility during activities specifically held for them at the respective senior citizens’ clubs. All participants are weekly-attenders of their respective senior citizens’ club at their local district. Digital invitation flyer was sent to older persons through WhatsApp. Forty older persons who met the inclusion criteria were contacted through phone call for their interest to take part in focus group discussions. Of the 40 participants, 25 agreed to participate. Among the reasons for non-participation were personal issues and overlapping in the schedule. Dates were scheduled for each focus group discussion. Number of older persons involved in each focus group discussions were limited to 6–7 participants considering age-associated physiological changes and to allow more effective interaction between participants [34].

Focus group discussions were conducted at the senior citizens’ clubs. A multipurpose room at the senior citizens’ club was used for the focus group discussion. The air-conditioned room is an enclosed space that is generally use for private closed discussions, meetings, events or guest speaker talks. When in use, the doors will be closed with a sign to indicate private event in-session. The room was checked by the research team prior to the focus group discussion for suitability. Tables and chairs were arranged in a rectangular shape, whereby all participants and moderator are able to see each other. Placard were also given for each participant to allow familiarity to the names of each participant. This is to ensure that every participant will be able to address other participants during the discussion, as well as to allow the research team to identify each participant during the focus group discussion. Light refreshments were provided prior to and after the discussion.

All focus group discussions were led by the same moderator (CN, a female physiotherapist with degree qualification and currently enrolled as a master’s student at one local university) and assisted by one post-graduate physiotherapy student. As the moderator was a master’s student, as part of her studies, she had some training in the conduct of focus group discussions. There is no relationship established between the moderator and the participants prior to the focus groups although participants were aware this research was being undertaken as part a higher degrees research study and that the moderator was the student. Written informed consent and demographic details were acquired prior to the commencement of the focus group discussions. Once the consent form is signed and the demographic questionnaire is completed, participants were verbally reminded that audio recording will be in use, before starting the recording in full view of all participants. The session began with a brief explanation of the study, including purpose of the research, proposed outcomes of the research, participants’ confidentiality and rights, and ground rules during discussion. Next, the focus group discussion was guided by a list of questions (Table 1) prepared in advance based on literature review [15] and opinions of professionals in geriatric field. The moderator prompted questions to participants as necessary in order to balance the level of each participants’ involvement in the discussion and guided the session according to the flow of the discussion. The assistant is responsible for notes taking throughout the discussion, which includes non-verbal interactions and general content. The entire discussion was audio taped. Audio-recorded data were imported to laptop and stored in an encrypted file. Only the research team members could get access to the data. Focus group discussions were carried out between December 2017 and April 2018 and each session lasted for an average of 1 hour and 15 minutes. Data collection ceased at the fourth focus group when no new information was generated.

**Table 1:** Focus group discussion guide.

1. What do you think about falls in older persons? (probe: how falls can affect older persons)
2. What do you think can cause falls in older persons?
3. Can you tell me what you do to prevent falls in your daily life?
4. Can you tell me what do you do after a fall? (probe: how to get up off the floor, how to get help)
5. What type of website characteristics do you prefer from a website? (probe: text size, text style, colour, design, use of images/video, navigation, page organization) Existing health websites were shown to the participants for their feedback on the website characteristics.

### Data analysis

Initially, the FGD data were transcribed verbatim by the researcher (CN) and another independent individual listened to the audio recordings and checked with the transcripts to determine transcriptions accuracy. Following that, the transcripts were imported to NVivo software for inductive thematic analysis [35, 36]. This involved researcher familiarization with the transcripts, and generation of themes, categories, and codes. Field notes taken by the assistant were used to supplement the verbal data and to provide context to each focus group.

The research team cross-checked the generated codes, categories, and themes. Any disagreements about coding and themes were discussed until consensus was achieved. As means of enhancing rigour and trustworthiness of the research process, a range of strategies were used. These include adherence to a focus group guide, audiotaping the focus groups, transcribing verbatim which was verified by an independent individual outside of the research team, cross checking the data analysis between the research team, and member checking [37–39]. All data were de-identified to promote trustworthiness of the data analysis process as well.

## Results

A total of 25 older persons were involved in this study. Four focus group discussions were conducted, three consisted of six participants each and one consisted of seven participants. Participants’ demographic details are as shown in Table 2. Majority of them were females (n = 20), in their 70s, and of Malay or Chinese ethnicity (n = 10, n = 11). Most of them had prior fall(s) (n = 21) and considered themselves at risk of falling (n = 15). Participants predominantly have basic computer skills (n = 17), staying with one or more people (n = 21), completed their tertiary education (n = 17) and self-reported good health status (n = 18).

**Table 2:** Participants’ demographic details.

Characteristics	n (%)
Gender	
Male	5 (20)
Female	20 (80)
Age	Mean 72.08 (range 61–83)
Ethnicity	
Malay	10 (40)
Chinese	11 (44)
Indian	4 (16)
Computer literacy	
Yes	17 (68)
No	8 (32)
Falls history	
Yes	21 (84)
No	4 (16)
Living status	
Live alone	4 (16)
One other person	5 (20)
Two or more other people	16 (64)
Education	
Secondary	8 (32)
Tertiary (non-university qualifications, university certificate, bachelor’s degree, master’s degree)	17 (68)
Self-rated health	
Excellent	1 (4)
Very good	3 (12)
Good	18 (72)
Fair	3 (12)
Self-perceived risk of falling	
Yes	15 (60)
No	10 (40)

Four themes emerged from this study. These themes are ’Perceptions of falls in older persons’, ’Actions taken when falls occurred’, ’Perceived prevention strategies for falls’ and ’End user requirements for falls educational website’. Table 3 details the emerging themes and categories.

**Table 3:** Themes and categories emerged from the analysis.

Themes	Categories
1. Perceptions of falls in older persons	Thoughts about falls
	The impact of falls on older persons
	Factors that result in falls
2. Actions taken when falls occurred	Immediate actions to reduce falls impact
	Getting up from the floor
	Ways to get help
3. Perceived prevention strategies for falls	Physical activity
	Home hazard modifications
	Footwear
	Nutrition
	The use of mobility aids
	Self-initiated precautionary approaches
4. End users’ requirements for falls educational website	Presentation factors
	User control

### Perceptions of falls in older persons

#### Sub-theme: Thoughts about falls

Participants held mixed views regarding falls. Some perceived falls in older persons as a preventable event, while some perceived it as non-preventable. Participants expressed their helplessness when discussing about falls. One participant even regarded fall as the will of God as it may still occur regardless of taking all necessary precautions.

*“Ageing is normal*, *falls is not necessary*. *It*’*s not normal to fall as you grow older*. *You more likely to fall is*, *not that you*, *old people have to fall*.*”*

*Participant 1*, *FGD 1*, *Chinese female*, *79 year old*

*“I am trying to think that everything goes in cycle*. *As a baby*, *you grow up*, *you fall*. *We always tell our children*, *falling is part of growing up*. *So when you reach a stage*, *falling is part of growing old*. *So I am trying to recall*, *is there any old person who has never fallen*. *All old people would have fallen at one time or another*. *So I think it is part of ageing oh*. *Because it*’*s a cycle*.*”*

*Participant 6*, *FGD 1*, *Chinese female*, *73 year old*

*“I think it is very difficult to prevent falls*. *It can happen*…*the*…*because*…*we have to accept that falling is like accident*, *and it is very difficult to prevent accident happen*.*”*

*Participant 2*, *FGD 1*, *Chinese male*, *77 year old*

*“But if with all these carefulness*, *if you want to fall*, *I think that*’*s the act of God*. *We can*’*t prevent God*’*s will*.*”*

*Participant 5*, *FGD 4*, *Malay female*, *70 year old*

#### Sub-theme: The impact of falls on older persons

The impact of falls stated by participants included injuries such as fractures, dislocations or head injury, and immobilization. Psychological effects consisted of fear, loss of self-confidence and depression following a fall. These psychological effects were seen to cause reduced social interaction, increased dependency, refusal to walk or move and reduced quality of life. One participant mentioned death as a result of fall.

*“Head trauma is the worst*, *you know*, *when you fall*.*”*

*Participant 5*, *FGD 1*, *Chinese male*, *69 year old*

*“Through what I observe and experience*, *people falls*, *some of them*, *within*-*within my family*…*Most of them when they fall*, *they either crash their backbone*, *crack their backbone*, *crack their hip bone*, *and err*…*what*’*s that*, *fe---femur*. *When you fall*, *you hit something*. *This*-*this happened to families*…*members of my family*.*”*

*Participant 1*, *FGD 4*, *Malay male*, *74 year old*

*“Basically*…*for me*…*the biggest*…*that*’*s the biggest worry*, *I do not want to lose my confidence in myself*, *that means I don*’*t want to get up*, *I don*’*t want to walk*, *I don*’*t want to do anything*.*”*

*Participant 6*, *FGD 3*, *Indian male*, *73 year old*

*“Psychologically*, *like the fear might also build up for you to move around also*.*”*

*Participant 3*, *FGD 2*, *Indian female*, *64 year old*

#### Sub-theme: Factors that result in falls

Participants mentioned a number of factors that may result in falls. For example, various medical conditions, consumption of too many medications and side effects of particular medications.

*“You will be surprised ah*…*even taking medications*, *especially the high blood pressure medication*. *Sometimes when it is too much*, *your pressure goes too low*. *My wife*. *Took the medicine*, *the next thing*, *I saw her on the floor*.*”*

*Participant 2*, *FGD 1*, *Chinese male*, *77 year old*

In addition, participants frequently cited personal risky behaviours such as being in a hurry, or performing hazardous actions as the cause of fall. Also, active pets or grandchildren can pose risks to fall in older persons.

*“And also having pets in the house can also pose the danger*. *Pet is running all over*, *and if they see the grandma they are so happy*, *they can jump on the grandma and that*-*that may also dangerous*. *And so are grandchildren*. *Grandchildren also very*…*you know*, *they are active*, *they run all over*.*”*

*Participant 2*, *FGD 2*, *Chinese female*, *66 year old*

Environmental hazards provoked in depth discussion among the participants. Slippery floor, loose or improper rugs and mats, steps, insufficient lighting and cluttered environment were reported to be dangerous to older persons.

*“I noticed a lot of houses they have floor mats or rugs outside the bathroom*, *or anywhere as you walked in*. *These are very dangerous also*. *You stepped on it*, *and you zupp*.*”*

*Participant 1*, *FGD 1*, *Chinese female*, *79 year old*

*“That*’*s also caused a lot of falls*. *Some don*’*t use proper rugs*. *They use old clothing*, *bed sheet*. *That*’*s worst*. *When you foot get caught in the cloth*, *then you tumble*.*”*

*Participant 1*, *FGD 1*, *Chinese female*, *79 year old*

Besides this, participants also raised attention to outdoor hazards, focusing mainly on street environment. Potholes, damaged concrete drain covers, root heave, uneven surfaces due to poor pavement maintenance were highlighted by participants.

### Actions taken when falls occurred

#### Sub-theme: Immediate actions to reduce falls impact

Participants emphasized on the importance of early recognition of a fall and the ability to recover balance or to avoid severe injuries due to a fall.

*“So when she fall ah*, *she said if I fell like normally*, *I would have knocked my head and would have got a haemorrhage or whatever*. *So she fell*, *and then she lifted up her head*. *So when she fall*, *her head didn*’*t touch the ground*.*”*

*Participant 3*, *FGD 1*, *Chinese female*, *72 year old*

*“The very important thing is*, *the moment you feel you are not steady*, *like not very balance*, *always recall in your mind is*, *you squat down*. *I think that*’*s what my wife did*.*”*

*Participant 2*, *FGD 1*, *Chinese male*, *77 year old*

#### Sub-theme: Getting up from the floor

The importance of remaining calm after a fall was indicated by all participants. This was viewed as the most important first step. Lacking of strength, knee problems, and do not know how to get up were stated as the reasons of not being able to get up or needing help to get up from the floor after a fall. All participants pointed out that inappropriate ways of assisting older person who is on the floor after a fall was dangerous and might cause further injuries.

*“I think when a person fall*, *you know*, *try and collect your mind*, *don*’*t be frighten*, *and don*’*t be embarrassed*. *Don*’*t like the minute you fall*, *you want to get up*. *Don*’*t care what people say*, *don*’*t care*…*calm yourself down first*. *I think to remain calm is very very important before you do the next thing*…*”*

*Participant 2*, *FGD 2*, *Chinese female*, *66 year old*

*“People who wants to help you is the most dangerous*. *Because when I slipped from the massage bed ah*, *quite high you know*, *and she dint know I injured*. *She pulled my hand*, *on*…*pull my injured hand*, *you know*, *that could have caused the fracture to be worse*.*”*

*Participant 3*, *FGD 1*, *Chinese female*, *72 year old*

#### Sub-theme: Ways to get help

Participants commented that mobile phone, bell, whistle or emergency button could be used to summon help in the case of falling. Shouting out loud was also viewed as a useful alternative to get someone’s attention.

*“You cannot get up la*, *so might else lie down lo*. *And wait for people to come*……*So*…*I think about 10 minutes*, *people passing by*, ’*Ah*…*help*! *Help*! *Help*!’ *and then help me up*.*” Participant 5*, *FGD 2*, *Chinese female*, *79 year old*

### Perceived prevention strategies for falls

#### Sub-theme: Physical activity

All participants mentioned about physical activity as a method to prevent falls. Among the physical activities perceived to be useful were Tai-chi, Qi Gong, balance exercises, strengthening exercises, stretching, walking and general body movements.

*“I*-*I do Qi Gong*, *not really consistently*, *regular*. *But I know it*, *if I can*’*t go to the field and do it*, *I do it at home*. *Even in the room I can do Qi Gong exercises*. *But I try to do it as often as I can*.*”*

*Participant 1*, *FGD 4*, *Malay male*, *74 year old*

#### Sub-theme: Home hazard modifications

Home hazard modification as a strategy to prevent falls triggered considerable discussion among the participants. Suggested home hazard modifications included having adequate lighting, installing handrails, avoiding having steps, use of non-slip mats/rugs, clearing clutter from the floor, ensuring walking surfaces are dry, sticking contrast tape on the staircase and replacing squatting toilet to sitting toilet.

*“Hand railing is the most important thing when you have aged person in the house*. *That*’*s the most important*.*”*

*Participant 5*, *FGD 1*, *Chinese male*, *69 year old*

*“At home is*, *especially the floor for the older people*. *Even the rug ah*, *you need to make sure the rug is not slippery*.*”*

*Participant 2*, *FGD 1*, *Chinese male*, *77 year old*

*“The hazards in the house*, *ya*, *like wires ah*, *normally people will tape it up isn*’*t it*, *like you go to the shopping mall*, *so maybe if cannot avoid*, *then maybe you tape it up or you put it behind the cupboard and all that*.*”*

*Participant 3*, *FGD 1*, *Chinese female*, *72 year old*

#### Sub-theme: Footwear

Participants mentioned proper footwear to prevent falls. Shoe characteristics such as enclosed, easy to put on, lightweight, comfortable, well fitted, sturdy, low heeled and non-slip shoe sole were highlighted.

*“Ideally*, *no need to tie lace*, *because if you tie lace*, *then you got to bend down you know*, *just something that can slip on but wrap around*.*”*

*Participant 2*, *FGD 2*, *Chinese female*, *66 year old*

#### Sub-theme: Nutrition

Some participants stated the role of nutrition in preventing falls. Balanced diet and healthy eating were perceived as important. Water, protein and fruit intake were discussed. The beliefs about how certain food should be consumed and the benefits of traditional herbs also emerged in the discussion.

*“So they have to find something to*-*to help increase the bone mass gain*. *This is a simple thing*, *the Indian have been practicing it*, *you know*, *this moringa powder*.*”*

*Participant 2*, *FGD 3*, *Malay male*, *63 year old*

*“And then err*…*we been practicing*, *we take fruits in the morning*, *before anything else*, *before anything else*, *take the fruits on empty stomach*, *and then let it for sometimes*, *I think about half an hour at least*, *then you go for your meal*, *whatever you want to take later*. *But fruits first*, *don*’*t take the fruits later*, *there is no effect*.*”*

*Participant 2*, *FGD 3*, *Malay male*, *63 year old*

#### Sub-theme: The use of mobility aids

Some participants agreed that walking aids can be used as a support to steady oneself. Although, a few participants provided examples of walking aids such as walking stick and walking frame, they appeared to be uncertain about the best selection.

*“Especially you are walking*, *and err you want to go*…*to the shop to eat*, *or to get something from the sundry shop*, *you have a step or two to climb*. *So if you are not very steady*, *so it is better to hold a stick*, *you know*, *that helps you*, *I will called it my third leg*.*”*

*Participant 1*, *FGD 1*, *Chinese female*, *79 year old*

*“Because the walking stick is not stable*. *So it*’*s always the better the walker*. *It (walking stick) helps to certain age only*. *It helps to certain age*.*”*

*Participant 5*, *FGD 1*, *Chinese male*, *69 year old*

On the other hand, participants also discussed some factors that may hinder the use of walking aids among older persons. All factors mentioned were associated with the stigma towards the use of walking aids. One participant pointed out that one of her peers is using an umbrella as a substitute instead of walking stick due to embarrassment.

*“My sis in law also the same*. *Ask her to use*, *不要 (cantonese*: *“don*’*t want”)*, *why*, *不好看 (cantonese*: *“it does not look good”)*, *我说你跌了就好看 (cantonese*: *“I said when you fall then you look good”)*.*”*

*Participant 1*, *FGD 1*, *Chinese female*, *79 year old*

*“They cannot accept that they have grown old*.*”*

*Participant 6*, *FGD 1*, *Chinese female*, *73 year old*

#### Sub-theme: Self-initiated precautionary approaches

This method of preventing falls received most comments and included walking strategy, practicing safe behaviours and being aware of falls.

Participants perceived walking slowly and carefully, walking on a safe path and looking ahead while walking as some of the strategies to prevent falls. In addition, one participant indicated that she believed landing with the toes first can help to grip the floor which then prevent falls from happening.

*“When I walk*……*I choose the place where it is safe for me to walk*. *Uhm*, *if there is pavement*, *and then there is a roadside*. *Do I take the risk of going on the roadside with cars going by or on the pavement which is un*-*uneven*. *Ya*, *so these*-*these kind of rules you should set to ourselves*…*”*

*Participant 1*, *FGD 4*, *Malay male*, *74 year old*

*“No*, *the floor is wet*, *you cannot land*…*This is a lesson for everybody*, *when you want to walk into a wet room*, *never go with your foot*, *heel (pointing at her palm of hand)*. *We are taught to walk*, *the correct way to walk is heel*-*toe*-*heel*-*toe*-*heel*-*toe*. *So you land on your heel*, *your slipped*. *You must land on your toes*, *then you can grip the floor*.*”*

*Participant 6*, *FGD 1*, *Chinese female*, *73 year old*

Examples of safe behaviours were holding onto someone or something for support, use appropriate tools or equipment to help with activities of daily living, staying in a familiar space, avoid sudden movements and rushing.

*“Most importantly*, *when they are old*, *let them stay in one place for long*, *not changing places*. *Yes*, *these are the thing*, *so that they know how to move*, *how much longer*, *how much height*…*all these things (in the house)*.*”*

*Participant 3*, *FGD 3*, *Malay female*, *61 year old*

*“So wherever I go*, *anywhere*, *I always have somebody to be with me*. *So that anything I can get hold of my grandchildren ke*, *my son ke*, *anybody la*, *my husband ke*, *to accompany me to cross the road*.*”*

*Participant 3*, *FGD 4*, *Malay female*, *77 year old*

*“For me*, *I have several rules*. *Several rules*. *1*, *when I get up from bed*, *I don*’*t get up straight away*. *There is always that 1 minute*, *2 minutes*, *before you move*, *before you get up*, *and then get out of bed*, *and then go to the bathroom*. *So it takes about 2 minutes before you reach the bathroom*. *But ah*…*the other rule is erm*, *no sudden change of*…*movement*.*”*

*Participant 1*, *FGD 4*, *Malay male*, *74 year old*

Moreover, being aware of falls also play a part in preventing falls. From participants’ perspective, being extra careful matters for older persons.

*“Like*, *ok*, *now we are going up the staircase*, *we know that*, *that can be a risk*, *at night*, *so you want to be very very cautions*. *Then going up even ladder*, *you want to climb up to reach something high*, *so you know you might*-*might have a fall*, *so you have to be careful*, *in what*…*the best whatever you can do*, *you do*. *That*’*s all*. *After that*, *is not in your hands la*.*”*

*Participant 3*, *FGD 2*, *Indian female*, *64 year old*

*“So you have more extra careful*, *so take every precaution not to have another fall*.*”*

*Participant 2*, *FGD 2*, *Chinese female*, *66 year old*

### End users’ requirements for falls educational website

#### Sub-theme: Presentation factors

Under this category, participants’ desired features were large font size, title and logo that attract users and colour contrast between background and text. Colourful website was viewed as one of the attractive features. Nevertheless, participants were inconclusive on what colour to be used on the website. One participant mentioned that decorative styles of typeface should not be used in a website for older persons as it challenges text readability. Apart from that, proper spacing and use of capital letters were commented. Moreover, participants also preferred consistent website layout and appealing website design.

*“Larger font for the old*.*”*

*Participant 6*, *FGD 1*, *Chinese female*, *73 year old*

*“Normally we use black against white*, *but you can see clearer*. *If use blue tu*, *you cannot see*.*”*

*Participant 2*, *FGD 4*, *Malay female*, *61 year old*

Special emphasis was placed on website headings. Participants stated that headings must be larger and in bold font, highlighted and come along with images.

Looking into the aspect of information presentation, participants suggested short, precise and direct sentences, minimizing the use of words, use numbered or bulleted list, and split different information into pages. The use of simple language was also highlighted.

*“And then I think you need to put it in point form which you can*…*you know*, *causes of falls or prevention of falls*. *If you want to show methods of prevention of falls*, *it should be like 1*, *2*, *3*, *4*.*”*

*Participant 6*, *FGD 4*, *Indian female*, *77 year old*

*“But break up into several pages*. *So when you concentrate one page*, *you have just one item in your head*, *and you look at it and understand*. *And the next item comes in the next page*.*”*

*Participant 1*, *FGD 4*, *Malay male*, *74 year old*

*“And then the language also*, *should be*…*must be simple*.*”*

*Participant 4*, *FGD 3*, *Malay female*, *62 year old*

All participants particularly highlighted on the use of images, illustrations or videos to deliver information. Additionally, the use of images with relevant text input was also indicated and accepted by participants.

*“And there was a picture to help with*, *it cuts down the border*, *picture always speaks thousand words*.*”*

*Participant 6*, *FGD 1*, *Chinese female*, *73 year old*

*“All of us want that you know*? *We want the picture to give us the message*.*”*

*Participant 3*, *FGD 2*, *Indian female*, *64 year old*

#### Sub-theme: User control

Participants demanded navigation buttons such as ’Page up’, ’Page down’, ’forward’, or ’backward’ to ease the website navigation.

*“You*-*you have the arrow to go forward*, *backward*, *forward*, *backward*. *You can use arrow or you click on the arrow*, *and the next page come up*. *Or click back arrow or forward arrow*, *you know*, *that*-*that sort of thing*.*”*

*Participant 1*, *FGD 4*, *Malay male*, *74 year old*

Insertion of audio function and option to increase text size were discussed and received mixed responses from participants. Some participants agreed to have audio function on the website while some disagreed. Reasons provided were mainly concerned with language spoken or reading speed. Similarly, only a few participants perceived the option to increase text size to be useful for older persons. On the other hand, majority demanded large font size without the need to manually adjusting the font size. All participants preferred to have multi-language option on the website.

*“And then we want the languages*, *all into Malaysian la*, *Malay*, *Indian*, *Mandarin*.*”*

*Participant 6*, *FGD 2*, *Chinese female*, *81 year old*

## Discussion

In this study, we explored older persons’ perceptions about falls and their desired educational website characteristics. Previous studies demonstrated that Malaysian older persons had minimum knowledge of falls [15, 40]. For instance, older persons tend to underestimate falls incidence, reluctant to use walking aids or modify home environment, practice of traditional treatment or home remedies for fall related injury, and the unawareness of medication related falls and prevention strategies [15, 40]. However, findings of this study showed a better knowledge of falls and prevention strategies among Malaysian older persons, even though negative stigma associated with falls still existed.

### Perceptions about falls

Older persons expressed mixed responses about falls. Falls were perceived as both avoidable and inevitable. Other studies concur with these findings. For example, majority of older persons in Sri Lanka and Ottawa, Canada held positive attitudes towards falls [41, 42]. Meanwhile, the negative perceptions of falls reported across studies included “falls as part of ageing”, “falls being uncontrollable and insignificant”, “refusal to acknowledge personal risk of falling” and “falls only happen to those who are older” [13, 15, 18, 43].

Notably, among participants in this research, falls that occur irrespective of the precautions taken, were seen as God’s will. Fatalistic beliefs about falls have been previously reported among older persons in South Asia [13]. Older persons of Chinese descendent, on the other hand, alleged suppositions that falls occurred by ’chance’ or ’luck’ [18]. The word ’lucky’ and ’god’s grace’ were used when sustaining minor injuries in falls among Malaysian older persons [15]. These beliefs led to the perceptions that fall incidents are non-preventable. Factors such as gender, age, educational level, and cultural background were associated with how older persons perceived falls [18, 41, 42].

Participants in this study expressed their concerns about the effects of falls, comprising of physical and psychosocial aspects. Similarly, previous studies showed that older persons were aware of the consequences of falls [15, 41, 44, 45]. In addition, participants mentioned a considerable number of fall risk factors, with greater emphasis on extrinsic risk factors. Remarkably, even if older persons were informed about some of the risk factors of falls, they did not take any necessary action to address them [44]. Some even ignored the possible risk of fall and continued performing high-risk activities [45]. These findings suggest that fall education and awareness are necessary to engage older persons in fall prevention. However, the association between perceived risk and fall prevention related behaviour may need further exploration.

It is noteworthy that participants emphasized on actions to minimize major falls consequences, namely head injuries. Suggestions provided included early recognition of falls possibilities, breaking a fall by lowering body’s centre of gravity and lifting or protecting the head. This indicates that older persons had substantial concern about falls-related injuries. The use of upper limbs and trunk flexion or rotation movements as a protective response against falls have been recommended [46, 47]. However, these protective responses are demanding for older persons due to its complex mechanism and age-related changes [47, 48]. Accordingly, upper limb strengthening, rotational falling technique and safe falling techniques are some of the proposed prevention interventions to reduce falls-related injuries [47–49].

Findings about older persons’ perceived falls prevention methods highlighted several strategies. These include physical activity and home hazard modifications, which are evidence-supported [50]. However, details of how much is known by older persons regarding these strategies is questionable and more studies are required. Previous studies reported that fall prevention methods commonly used by older persons consist of home modifications, safe footwear and usage of walking aids [41, 42, 51, 52]. These indicated methods are generally considered as external strategies, which refers to external aids or environmental modifications [51].

Besides this, several self-initiated precautionary approach in preventing falls were mentioned by older persons in this study. Such examples are ’adjusting gait pattern’, ’implementing safe behaviours’ and ’being alert to the possibilities of falls’. These findings corroborate with previous studies, whereby older persons [51, 53] frequently practiced behavioural interventions. These include being constantly self-aware, to watch out for fall risk, altering walking patterns, slowing down movements, avoiding hazards or risks, asking for assistance and refraining from activities which could cause falls [45, 51–54]. The tendency of older persons attributing falls to their personal falls risk factors could explain this finding [53]. Older persons considered ’taking care’ as the primary way to prevent all falls, despite the fact that falls are multi-causal [44]. This finding is worthy of attention because behavioural intervention like intuitive strategies, has lack of evidence, and if used alone, it would not be sufficient to prevent falls. One unique finding of this study was that current Malaysian older persons are staying together with their families and they preferred to stay in their own home rather than relocation, indicating strong social cultural influence among Malaysian older persons. This practice made them believe that companionship and familiarization with the living environment might reduce their risk of falls. Available studies on social cultural influence on living arrangement and environment in Malaysia are limited [55, 56] and more extensive studies are required.

Another point highlighted in this study is the role of walking aids in preventing falls. Divided opinions on the use of walking aids were noted. Older persons perceived walking aids as useful, but seemed indecisive about the types of walking aids. In addition, negative labelling associated with using walking aids were of particular concern to older persons. Considerable similarities were shown in the previous studies [45, 57]. Self-consciousness about the appearance of ageing was the primary barrier to the use of walking aids [45, 57]. Older persons were reluctant at first regarding adoption of walking aids, but this perception changed over time [45, 57]. The benefits of walking aids in enhancing confidence, independence, activity and participation superseded the associated negative stigma [45, 57].

Walking aids are usually prescribed to address gait and balance impairments that may cause falls. However, the use of walking aids itself may be a risk factor for falls [58]. This could be due to obtaining walking aids without consultation from related healthcare professionals, improper fitting, walking aids that lack maintenance and inappropriate ways of using walking aids [57, 59]. This again stressed the importance of falls education for older persons.

It is surprising that even though participants attributed falls to certain medical conditions and medications, none of them mention any prevention strategies targeting these risk factors. Similar results were demonstrated in previous studies, whereby older persons were unaware or less aware of the role of medication review and management in preventing falls [42, 51, 52, 54]. This indicates that there is a need to educate older persons about medical and medication related fall interventions.

Regarding website characteristics, participants prioritized presentation factors more than user control. Presentation factors in this study context refer to factors that create visual perception of users. Generally, this shapes the first impression of websites [60, 61]. A possible explanation for the less emphasis on user control is may be due to the lack of knowledge on website navigation.

### Preferred website characteristics

In our study findings, presentation factors discussed were focused on text readability, website aesthetics and content presentation. Text readability could be enhanced by using larger font size, appropriate typeface, ample spacing, careful use of upper and lower-case letters, colour contrast between text and background, and clear headings. These findings are fairly predictable as easily readable text can help older persons to overcome barriers imposed by age-related vision decline. Suggestions from participants are comparable to those reported in available guidelines [24–26]. However, recommendations on typeface weight and text justification being suggested in the guidelines were not mentioned by older persons in the present study.

In regard to website aesthetics, participants highlighted that it should be colourful, of appealing design and with a consistent layout. The latter two are the first thing that determine how users perceive a website and their decision whether to continue browsing or move on to another website [62]. Although a colourful website was preferred, selection of colour must be done conservatively considering older persons’ changes in colour perception [20, 25, 26]. Yellow, blue and green colour should be avoided when close to each other as older persons may have difficulty in distinguishing them [20, 24, 26].

For content presentation, participants desired short sentences, simple languages, organization of information and use of images or videos. Similar suggestions were highlighted in previous guidelines with additional emphasis on the style and phrasing of sentences [24–26]. Older persons’ preference for the use of images or videos in addition to text were also shown in previous studies [22, 28]. As reported by Schenkman & Jönsson [60], more illustrations can produce first impression among users. However, only relevant images should be inserted in the website [24, 26] to prevent confusion among older users [28]. Despite the preferences for video, older persons in our study did not elaborate on its use. However, the issues of unfamiliarity with the use of video or absence of user instructions have been raised by Nahm et al [22].

Availability of navigation buttons and multi-language options were the key user control requirements emerging in our study. Easy web navigation incorporating step by step navigation procedures, use of single mouse click, providing location of every page, large buttons with text labels, careful use of pull down menu type, and navigation buttons such as ’previous page’ or ’next page’ are some of the recommendations in the website design guidelines [24–26]. These features can aid in reducing cognitive, dexterity and visual demand in older persons when accessing the website. In view of the multiracial Malaysian population, multi-language option was perceived as important by older persons in the present study.

On the other hand, older persons in our study demonstrated conflicting responses to speech function and options to increase font size in the website. Based on the guideline for website design, speech function and option to increase font size may be inserted deliberately [25]. Besides this, text-to-speech function should consider the voice spoken and speed of reading, taking into account older persons’ changes in hearing and cognitive status [20].

Limitation of this study is that it involved community dwelling older person from an urban city in Malaysia. They were predominantly English speaking, highly educated and in good health. Thus, their perceptions of falls and requirements when accessing websites may not be applicable to older persons from rural areas and those with any motor or cognitive impairments. It is believed that older persons who have motor or cognitive impairments would have specific requirements pertaining to website interface. Nevertheless, this study included older persons of multi-ethnicity, with or without prior computer experience to ensure extensive views.

In conclusion, community dwelling older persons have mixed perspectives about falls and falls prevention strategies. Some differences were found between what older persons commonly think about falls and its associated prevention strategies, and what the evidence says. This highlights the importance of education in improving older persons’ knowledge on falls. In addition, older persons have unique requirements for website design, particularly in terms of colour and language, which need to be addressed when developing websites targeted for them. These findings also shed light on the unique needs of older people who are not from the Western world whose ethnic, cultural and social differences may require careful consideration.

## Supporting information

S1 File (DOCX)

S1 Checklist COREQ (COnsolidated criteria for REporting Qualitative research) checklist. (PDF)
