# Supplementary material for: Malaysian older persons’ perceptions about falls and their desired educational website characteristics: A qualitative study
Source: PLoS One. 2022 Jul 7;17(7):e0270741. doi: 10.1371/journal.pone.0270741 (PMC9262233; doi:10.1371/journal.pone.0270741)
Supplement: S1 File — (DOCX) [file pone.0270741.s001.docx]

**FGD Transcript 1**

**C=Moderator**

C: Good morning and welcome to our session. We would like to extend our gratitude to everyone who have attended today and share your views on the development of this web-based falls prevention education program. Your willingness to take your time and participate in this focus group discussion is highly appreciated.

My name is Cxx, I am a master student under the physiotherapy program, at UKM. I am currently doing my master under the guidance of Associate Professor Dr Dxx, Associate Professor Dr Txx, and Dr Sxx. We were pleased you could all join us today. I am the moderator for this focus group discussion, and my friends, Jxx, will be helping to taking notes and record the conversation. We will be going to be tape recording today so that we don’t miss any of your comments.

I am conducting a project on the development of a web-based falls prevention education program based on the needs of Malaysian older adults and evidence-based practice guidelines. The reason why we are gather here is to carry out a focus group discussion to understand what are the needs of older adults with regards to this website. The discussion will cover the area, such as the content of the website, the presentation of the website, and the usability of this website. The outcome of this focus group discussion will be integrated while we developing the website.

Before we start, there are a few rules that I would like to inform to all of you.

1. First of all, if you have a handphone, we appreciate if you can turn it into silent mode. (*Pause: everyone started to silent phones)
2. The most important rule is that only one person speaks at a time. There may be a temptation to jump in when someone is talking, but please wait until they have finished.
3. There are no right or wrong answers.
4. You do not have to speak in any particular order.
5. When you do have something to say, please do so. There are many of you in the group and it is important that I obtain the views of each of you.
6. Many people have different views. So feel free to comment even if your thoughts and ideas are different from what others have to say.

C: Does anyone have any questions?

C: Ok, let’s begin.

C: First of all, I’d like everyone to introduce yourself. (Interrupted by noise) We have name tags with your name on. (Interrupted by noise) Can you tell us your name, where are you from, what you work as previously, and have you experienced a fall before or do you know anyone who had a fall.

C: Ok, I will start. I am Cxx, I am from Taiping. (Interrupted by noise)

CT: Mine?

KH: Yours.

CT: I don’t know. Sorry. Sorry.

C: (continue) Ok, I am currently doing my master at UKM. I have completed my degree in Physiotherapy at INTI International University. Ok, we continue with Sis S.

S: I am S. I was a teacher, I am now a retiree.

C: Have you experienced a fall before?

S: Ya, couple of times. Bad one, hit on my head.

C: Sis SH?

SH: Ya, I am OSH. I am a retired nurse. Hmm, I currently residing in KL. PJ rather. But I was from Taiping, ah…Kuala Lipis, Alor Setar. My family are there. But I am here.

C: Do you experience a fall before?

SH: Oh, I had a…a few falls.

C: Ok. Bro KH?

KH: My name is LKH. Retiring. Hmm...my previous...Before I retire, I was doing mostly engineering work. Ah…with regards to fall, actually I have fallen from bicycle also. (everyone laughed) And the last fall was, I was in a hurry, was carrying something, two handful. And actually, I tripped on the road. But err…because I was able to observe that I was falling.

KH: No no, I was totally out. I was carrying the watermelon. I was able to break the fall. Then…as I was falling, I could observe and I turned my head away. So I got bruises there, in chin, that’s all. Otherwise I could have hurt more ah when I face down.

C: Bro J?

J: JV. Retired, 13 years now. Was with the technical side in telekom. Ah…I ride a bike all the time, so…just…you know how reckless they are, so one rider crashed into me, so I fell, fractured a rib. So, I guess my fall could be riding-related la.

C: Sis CT?

CT: Ah, ya. I am CT. I am housewife. Erm...I had a fall last year. It was err…actually not being mindful. I missed a step, and I fall flat. Other than that…That’s why it is also good that, you know, now I am very very careful with the steps, make sure it would not happen again. Other than that, quite ok.

C: Sis Y?

Y: I am BY, from Melaka, now stay in KL. I was…err… a retired medical social worker from UMMC. I had a few falls, but I don’t think that it is due to my carelessness. Once was I was in China, on a massage bed, and they didn’t have, it’s a slippery bed with no towel on it, you know? So when, there are…maybe some powder and the whole, you know, there’s no bed sheet on the bed, there’s a towel, so the towel slipped, so I also together fell with the towel on the floor. And I broke my shoulder.

(*everyone oo-ing / sound of shock)

Y: (pointing at her right shoulder) Here here, quite a bad one. And another one was, I was on medication for helicobacter pylori, that’s the bacteria in the stomach. The very nasty. So I was on two medications. And I was quite dizzy, you know…

S: Drowsy. Antibiotic. Very strong dose.

Y: First day coming back from MC, two months MC. I think. First day itself I fell in the hospital. Because it was raining, and the foyer was wet, you know…so I tripped over a chair, the stand sticking out, so like you are a bit dizzy, so I tripped on the leg of the chair and I fell, and I broke my wrist.

C: Ok, now let’s get started with the questions. The first one, tell me this, what is falls in general?

CT: What is?

Y: Falls.

C: What is falls in general?

Y: (repeat) Falls.

S: What is falls in general?

S: Fall mah fall lo.

J: You mean the definition of a fall?

C: Ah yes, what you think…

J: The definition of a fall, what caused it?

C: Uhm, just…

(everyone): No.

S: What is a fall la.

C: Yea, what is a fall?

S: The definition of a fall.

S: Losing your balance? (silent for 5 sec)

C: Anyone? (silent for 5 sec)

C: What do you think of falls?

J: I guess err, fall is, what she said, is correct, is losing your balance.

KH: Or out of control, you are not able to control.

J: Involuntary.

KH: Ya.

C: That means by accident?

S: It’s always accident, never on purpose.

CT: Ya. Who will want to, you know, purposely fall?

S: But you still can mindfully fall.

CT: Ah?

S: Serious.

SH: I say ‘I am falling. I am falling. Falling. Falling.’ (laughed)

CT: Mindfully? Beyond your control?

S: I said it because I experienced it. I was going to enter the wet floor. I looked down and said: ‘Careful. Fall. Wet floor. Careful. Wet floor.’ One leg go in, zupp, you go.

SH: Careful and yet you fall? (laughed)

S: Yes. So mindfully you are aware, you are aware that you are going to fall, your hand can’t grip the door, you are standing at the doorway. If you are younger, you can hold on onto the door and prevent a fall, you know. Once you are old, you know you are going to fall, your hands ah…no action comes oh. You go down, so when you go down, I was bleeding, so I was telling myself, 像有鬼一样(hokkien) superstition nah.像有鬼推你(hokkien) because you are so mindful what, you said careful, wet floor, still you fall. But when you recovered and you analyse, it is still your own mistake.

C: Ok, so what do you think, Sis Y?

S: No, the floor is wet, you cannot land…This is a lesson for everybody, when you want to walk into a wet room, never go with your foot. Heel (pointing at her palm of hand). We are taught to walk, the correct way to walk is heel-toe-heel-toe-heel-toe. So you land on your heel, your slipped. You must land on your toes, then you can grip the floor.

Y: No, never got even stepped into the wet floor. You go round the wet...

S: You have no choice, it’s a bathroom. No water, water rationing. So you cannot go into the shower and bath, you need to go out bath in the hand basin, so the whole floor is wet. Mindfully you still fall.

C: Sis Y?

Y: Sorry, you were saying something? Never mind. You say first. (asking sis CT)

C: Sis CT?

CT: I forgot…

Y: My sis when she fell, she was mindful, so that she didn’t…because she was in the market, and don’t know how you know there was a slight ramp like that, so there was some sand, and then the sand make her, just slipped like that you know, and she fell. Luckily she was a PE teacher before, now retired. So when she fall ah, she said if I fell like normally, I would have knocked my head and would have got a hemorrhage or whatever. So she fell, and then she lifted up her head. So when she fall, her head didn’t touch the ground. That really save her from very**…**otherwise I won’t be talking to you right now. She might be just unconscious.

C: What do you think about falls among the older population?

Y: High, very high.

CT: Higher. The rate is higher.

J: Very prevalent. Very prevalent among the oldest. Because ah…really their senses are not all there. Balance. The footwear.

KH: You will be surprised ah…even taking medications, especially the high blood pressure medication. Sometimes when it is too much, your pressure goes too low.

J: Right.

KH: My wife. Took the medicine, the next thing, I saw her on the floor.

S: Not regulated.

KH: But I do not know how she landed on the floor. But she didn’t get any injury. Which means that she must have sense that, like dizziness, then she was able to control…and then…on the floor. And that happened. Because we didn’t know, you see. Sometimes the medication became too strong. So there’s one more danger on this.

CT: Especially people on the high blood pressure medication, when you get up, you are not supposed to get up and walk straight away. You got to, if you are lying down, you sat up on the bed first, for a while because when you get up, the pressure drops. Especially when you are on medication, then it will drops faster. Then when you get up, don’t straight away walk, you also got to get up, steady yourself first, before you walk. Because the pressure drops very fast.

C: Do you all think that falls can be prevent among the older population?

S: Yes, definitely.

C: Do you think that is a normal part of ageing?

S: Yes, normal part.

J: Yes, yes.

CT: Yes, ageing is normal. Because your balance is compromised as you aged.

J: Yes.

SH: Ageing is normal, falls is not necessary. It’s not normal to fall as you grow older.

J: Ya, we try to prevent it. That’s all.

SH: You more likely to fall is not that, you, old people have to fall. You just have to be more careful, because we are not that strong and steady anymore. So we got to balance ourselves first. So that takes some time. If you really in a hurry to get up and walk, then of cause you will fall. That is definite.

C: So Sis SH think that falls is not necessary to be happen when someone become older?

SH: Ya. Not necessary.

J: Yes, that’s true. We try to prevent it, as far as possible.

Y: No. Even young you also need to prevent falls. It’s just that old people, sometimes, because the…this capacity is full already (pointing to her head). (laughed) (SH: saturated.) Think of so many things, you will become less mindful la. And then the senses, like our sight is not that good, hearing not that good.

SH: Muscles are not that strong. You are not so steady.

Y: Reflexes is also not…reflexes…

CT: Tends to lose our balance more.

Y: The reflexes, like usually fall, you just simply grab. But when you are old, might be slower, the reflexes. So that will put us at greater risk. It’s not that old age, we can fall, it’s just that our senses are slow already.

J: it’s also because as we age ah, there are lots of aches and pains ah…and we are compensating for it ah…so this affects your overall sense of balance…It does…

Y: And when you are older, you need more medications. And the medication, ya, side effects…Like the medicine that I was on ah, if I was not on ah, I would have…

C: Sis S?

SH: The elderly are not so active. So their muscle tone is less, is reduced. When you balance yourself, you take more afford to balance, so if you don’t have the afford to balance, definitely you will fall. So it is got to do with your musculature, your strength, you know, that kind of things.

S: I am trying to think that everything goes in cycle. As a baby, you grow up, you fall. We always tell our children, falling is part of growing up. So when you reach a stage, falling is part of growing old. So I am trying to recall, is there any old person who has never fallen. All old people who have fallen at one time or another. So I think it is part of ageing oh. (laughed)

J: Yes, yes.

S: Because it’s a cycle.

C: Ok, the next question. Please tell me what do you know in details about falls in older adults? Where falls can occurred? The location…of falls?

Y: Home.

SH: Bathroom. Kitchen.

CT: It can…Actually it can happens anywhere. Anywhere, but it depends on…you know.

S: Places more prone la. Steps. Steps, when you have children in the house, the toys are everywhere.

CT: That’s why the active animals, you saying, the dogs and the cats, they are running around.

SH: That’s I said the fellow jump on me, I will fall backward. The fellow is getting so big.

CT: Which one?

SH: The dog. The strayed puppy. Getting very big already. When he jump on you, literally land on you, it’s so heavy.

S: Before I forget, can I share this ah? I read somewhere that old people are more prone to fall because we don’t walk in big strides. Mini mini steps we take. And because of this our pelvic is locked in, so when you want to make certain turn, you are already locked in, you fall. So the advice is, when you walk, take big steps.

CT: That brings me back, I meant I remember, this Japanese doctor, the one o, somebody going around in the apps, he is like taking the stairs, two, double. But it’s quite difficult you know sometimes. If it is near…I meant closer, then it is easier la. If it’s the wider one r, we really feel the stretch. But I think it is also a good exercise, you know.

C: Can everyone tell me the location that you think that most falls can happen? One location?

KH: Staircase.

C: Staircase. Can you explain more?

KH: Missed steps. Not mindful. My daughter fell 10 steps.

CT: Oh, 10 steps.

Y: Ya, one of my nephew also.

CT: She break any of her bones?

KH: Luckily no. Only she said like, I feel, I over-exercise one part of my body.

C: Err, bro J?

J: I think the slippery floor and footwear counts very much. The type of footwear, can cause a lot of falls.

C: Can you explain more on that?

J: I am actually talking on behalf on my mother who was 95. Not me ah.

C: Just share your stories with us?

J: So I am very careful on her footwear, how good the base is, I will look into it. Can be slippery the old one, have to throw it away.

S: Grip, must be a good grip.

J: Ya, must have a good grip, to fit well. Has to be well fitting. Of cause the next thing is the slippery floor. Never have a wet floor in the house la. You can have it, always the bathroom la.

CT: Bathroom la,不好(hokkien).

S: You know where to get a good socks, for old people ah?

S: At the bottom is,像有一粒一粒rubber这样的(hokkien), nine dollars a pair or eight dollars a pair.

C: Sis SH, where do you think falls can happen, most common?

SH: In the house.

C: In the house, like more specific?

SH: Hmm?

C: More specific?

Y: In what part of the house?

SH: One thing is slippery floor, wet floor. I two falls in the house, both also because of wet floor, (laughed) because the maid was cleaning the window, and then put the bucket there, then she moved to the next spot, this spot is wet, so I didn’t know, I was walking to the front, zupp, I went there, flying, twice.

S: Also caused by that…

CT: Ya, I also, I feel is also the…floor, then the toilet, I mean bathroom. That’s why I think, but it is our Asian culture what, we always wet, the Whites, I think their bathroom they are all dry, carpeted some, you know. Another thing…

Y: Sorry, let them finish first.

CT: So it could be, you know, because their country is, they are older, they can like that la…you know, otherwise we maybe can also look into that, but it is not so practical for us.

C: Sis Yen?

Y: Because our bathroom is small, and then the toilet is there, the shower is down there, in the same place. So I feel it’s very important for to have a shoe, sandal…No, no, no. It is not practical la, because our houses are small. Like apartments, they are small. What is important is to have a good slipper, with the good grip, in the bathroom, so like here, every time you go in, you wear the slipper. But never clog, don’t wear clog, don’t put clog. You know, the wooden…

CT: Oh. The wooden clog.

Y: Ya, there is no grip at all, deng, you can just slip and fall. So and also the rug, you know, the rubber mat, some bathroom there, we have that mat also. Because you know why bathroom is at more and more risky. You know, sometimes when, whoever washes the bathroom ah, whether maid, might not washed it clean and the soap remains, you know, so soap plus the water, can just slip very easily. While other parts of the house, it’s not sweeping well, soapy ah, if they don’t rinsed properly, that soap remains. That’s the most…

C: Sis S?

S: Market place.

S: Especially those big umbrellas that come out. They have these octopus.

(everyone): Oh

Y: That’s the one la. The dangerous part.

S: Many old ladies fall. Because they missed those octopus stand.

Y: Yes.

C: Just now some of you have mentioned that falls can happen when you slipped on the slippery floor, or you tripped over some toys on the floor. Any other activities or situations that might put you at risk of falls at home?

CT: The active pets.

C: Anything else?

SH: Actually, even the furniture also, you know. They are blocking your way, you know. You try to avoid it sometimes, you kicked, you kicked the foot of the chair, kicked the foot of the chair ah, you will tumble.

Y: Cluttered. The house should not be cluttered.

S: Once you are rushing around, you are bound to have this type of problem. So don’t rush.

J: You are right ah. The clutter. You have watch the clutter on the floor.

Y: Those unnecessary things around the house, you should throw away.

SH: I was in my friend’s house, the seating room is about half this size, they have this big rattan chair, you know, two seater, and single seater, they have one there, one there, I was sitting there, and the table in front, also have got this rattan this thing, this side also have a chair with a rattan foot, a huge one, I was coming up from there, I was sitting on the chair, coming up, and I kicked the…as I was negotiating, to come out from the corner, I kicked the leg of one of the chair. And I broke my toe.

CT: Aiyo…

SH: Broke my toe!

CT: It will takes you months to recover.

S: Toes hardest.

SH: Not a hard kick you know, just a slight kick.

CT: Because I think the bones is very…very brittle.

SH: And also the angle I think. Why my bone like that. I thought it wasn’t very painful because it was at the toe, very…at the tip. So I said, why my toes got cramp you know. Cramp already like that, so got to wait for it to straighten up. Eh, it won’t straighten up la. (laughed) Then I try to bend it, aiyo painful ah. Went to see the doctor, oh got a fracture.

C: Bro KH?

KH: Ah, your question is about the falling?

C: Yes, like what situations or activities that might put you at risk of falls?

KH: When you are in the any activities that are of high risk.

C: For example?

KH: Even if, you say, cycle, or you, err…very high speed, in motorbike, and things like that, you can fall.

C: If at home? Any activities?

KH: At home is, especially the floor for the older people. Even the rug ah, you need to make sure the rug is not slippery. You must have certain amount of grip ah, otherwise there’s a good chance to fall.

C: And then now, can you tell me how falls can affect someone? What are the consequences of falls?

S: Break your bones.

CT: You know, you can break your…you know, injury…you may have, like Sis Ooi, broken bones, you know, it can be very serious or it can be mild. But important thing is not to have any of these.

C: Injury. Err…fracture. Anyone else?

KH: Falls for maybe people who are already more than 70 years old and all that. My observation is, like my wife, she fell, she fell, the staircase, and after falling the staircase, you know, she got shingles, she got thyroid and parkinson’s. It all keeps on following.

Y: Illnesses.

J: A lot of factors.

KH: Actually, there’s not much observation been made to track that, people with that kind of age, and when you fall this things happen, happen, happen, following each. That’s what I observed. And actually, for many others, because people take to hospital then come back, you don’t think about it. But I think. When you…that’s why it’s so important when you hit the age of 70, 75 or what, if you fall, this chance of things happening like that, because our body cannot take too much stress, then the sickness comes.

J: Oh. It’s a snowball effects ah, what you mean? Snowballing effect ah?

S: Has a study been made on this snowballing effect? First time I heard, that’s why I am curious.

CT: Possible, these are all the, it could be a symptom, you know, and then, you know, it could be related.

S: Is there a research on it?

C: Like maybe the falls, if it lead to some head injury, then…

SH: I think other illnesses, I don’t think it’s related.

KH: She could have actually have some kind of a series minor stroke where you can’t track.

SH: Yes, yes, stroke yes.

J: TIA.

KH: When you talk about that, she didn’t lift the leg high enough, tripped at the, actually got caught at the edge. Some staircases you got to be very careful you know. Erm…they have this aluminium thing at the edge there. And her heel got caught. Got caught. Then she went off balance and topple down, hit the head. And she went through that stress, five days later, got shingles you know. Because you went through that, you have the stress, the shingles cannot catches up with you.

S: So the stress leads to shingles, and then?

KH: Thyroid.

Y: Eh. Not related what.

S: Thyroid also stress ar?

KH: You will be surprise. Because you thyroid.

CT: Your immune down. I think.

KH: Then come back, unable to remove. And you know, the thyroid appearing, it was lucky, when you see the neck big. Her case ah, you don’t see the neck that big, it goes inside.

SH: Oh. That’s the toxic type. There are two types. Thyroids there are two types. One is produce extra toxin, so it becomes toxic. Some they don’t produce enough, so they get the swelling of the neck. So there are two different types.

CT: Oh thyroid.

KH: Until the doctor said, yours is the king kong size already.

S: Aiyo. King kong size, that means you must be go on thyroxine la.

KH: No, first she go to get removed. Remove the whole thyroid. She is on thyroidism.

C: Sis Siew Hong. What do you think of the consequences of falls?

SH: Consequences of falls in elderly usually is fracture. The most common site is…

J: (Interrupted) Head trauma.

SH: Common site is neck of femur. The hip. Because err, say what you like, you touch 70, you bound to have some degree of osteoporosis. That means the bone is thinner, less calcium. So it is easier to break. The bone is more brittle. And for elderly people, very very common it is the hip.

S: That’s the weakest joint.

C: How, how do you think, what do you think falls can affect someone like, psychologically?

J: Yes, it does. It affects your self-confidence.

KH: Fear.

S: The fear comes in.

C: Anything else? Other than fear of falling and less of confidence?

S: Some older people don’t even want to walk. They don’t want to walk.

Y: Stay home. And they don’t want to go out.

Y: Can I add on the bathroom, why it is so prone. It is also because since it is slippery, there is no hand railing, you know, bathroom especially where elderly people stay, so have some hand railing, so when there is a fall, or when they go in, they can hold on to the…

J: Hand railing is the most important thing when you have aged person in the house. That’s the most important.

Y: Ya, like staircase.

J: Everywhere. Everywhere.

Y: Some staircases ah, the hand railing is so thick you know. So you cannot have a good grip, so that’s why, one friend ah, she said I was holding hand railing, but she said it’s so fat, that only half the grip, so she fell, and whole shoulder joint is…

CT: You meant the hand railing is so fat?

Y: Ya. Ya.

J: Some of them are…very, in hospital, because hospitals are big fat one.

Y: You know, for Asian hand ah, it’s not enough, for children’s hand also cannot. And also Asian hardly use the bath tub. You know, the hotel type. One friend she fell and knocked herself against the used bathtub, you know. And her head was bleeding and she stayed in hospital for few days. One of our Buddhist friend. CY wife.

CT: Oh, CY wife, Ixx.

Y: Yes, Ixx.

CT: Is it?

Y: She dint tell us. But she stayed in the hospitals for few days. Bleeding you know. So I mean, if you hit against the water basin, at least not solid and big, but big bathtub ah, it’s very very dangerous. And we hardly use it, it’s such for show only. Old houses got, but new houses don’t have. The hotel all very dangerous. You all have to be very careful. Especially you step in and out of the bathtub.

CT: But they have the hand grip.

J: Bathtub is very very dangerous.

CT: It’s not necessary for our culture. No la.

Y: Not necessary.

J: Walk in and dry. Must have a wet and dry part. That’s very important.

C: The next question…

SH: Sorry ah, I noticed a lot of houses they have floor mats or rugs outside the bathroom, or anywhere as you walked in. These are very dangerous also. You stepped on it, and you zupp.

J: You must have anti-slipped, underneath. You must have anti-slipped.

S: Anti-slipped. Otherwise…

J: Oh, it’s very dangerous.

SH: That’s also caused a lot of falls. Some don’t use proper rugs. They use old clothing, bed sheet. That’s worst. When you foot get caught in the cloth, then you tumble.

C: Ok, the next question. Tell me the causes that you are aware of that can lead to falls among the elderly.

Y: Oh we have done part of it.

C: Just now we have talked like, part of the environmental risk factor, like around the house. Any other causes that you are aware of?

J: Make sure whatever steps you have in the house, should be just about the maximum of one inch, or less.

Y: That’s most dangerous. If you don’t notice it, and you kicked it. You missed it.

CT: You have to put like sticker or what, the tape.

Y: That doesn’t help.

SH: You won’t look down and walk. Sometime by the time you come near it, you already tripped.

KH: For that part of one inch, (J: or less than one inch) I think even one inch you can trip and fall.

J: Oh. Definitely. Definitely.

CT: Not only one, it’s not level also. Can be half you know.

J: How to get a house perfectly level?

S: That’s why nowadays they don’t make the draft of one inch. They let it be gradual. The modern day is gradual.

Y: And also no footwear all over the doorway. Because I know one of the friend. She is blind. She’s about almost 80. So one day, she just, she didn’t fall, you know, she just stepped on to a shoe, off-balance and she fell, broke her hip.

J: You have to look into all aspects. Maybe your caregiver, I know it, because I am in for it 30 years now.

CT: Your mum with you for 30 years?

J: Ya.

?: How old is she now?

J: 95.

CT: Her hearing is no good. But others than that she’s still…

J: A lot of things to do la anyway. Some good days, some difficult days.

Y: You know like places where public goes ah, with footwear, and then put on the floor, like some temples, you know, so the shoes are all over the place. You should have move to the side, so that you can go up to the staircase. And after a while, again it filled up. So I think maybe proper places to put footwear. But even then shoe rack, people don’t use. They still take out and…(SH: People’s conscious is not there) Tzu Chi is very very fantastic. You cannot see footwear one. They all…

S: Nalanda is the same. Don’t only say Tzu Chi.

Y: I know. I know. Even BGF now also.

CT: The cupboard, the cabinet. So everybody put in there.

Y: The shoe cabinet.

J: Whoever is taking care of the elderly, and all this functions la, you need a person, you cannot do it alone.

Y: Even you put the label, footwear, please put shoes on the shoe rack.

C: Any other causes of falls? Causes of falls.

S: I think we have covered.

J: If you looked through, and then, maybe you explain on it la.

S: Tell us what we have missed.

Y: Oh on the road. Can also. No potholes on the road. Especially on the pathway where people walk ah. Sometimes you suddenly see a hole you know.

S: That one must sue the government.

J: Malaysia and India standard are the same. Tokyo is all safe.

CT: No pothole. Actually…Sorry ah. If it’s shallow, it’s ok you know, some can be so deep, and…

J: Of course, when its steel and grating.

CT: It’s a hole, and the motorcyclist at night, that’s why some are being killed.

J: They died. All the time they died.

KH: The problem is that, now it’s not safe to walk on the pavers, you know. Especially the older area, because there are so many things going on, and they don’t repave the pavement smooth. And then another part is trees, the roots start upheaval on the pavers. My sister keeps on falling until she said I won’t walk there, I walked on the side. I walked on the side of the road. She takes the risk.

SH: Safer to walk on the road.

KH: She’s been falling and falling.

J: Hui yo…

Y: Another thing, they put the cover on the drain. But one friend ah, because the cover gets old and old and, there was a crack already. The cement covers, along the drains, the final person who steps on it, it’s already breaking, and she didn’t know. She broke and she fracture her leg. You step on it. It breaks.

J: Serious la that one.

J: Our pavements are very dangerous.

Y: They don’t maintain. Once they see crack, they should quickly remove and put a new one already.

C: What type of medical conditions that you think that it will causes falls?

J: Old age.

Everyone: Medical conditions.

CT: Dizziness.

Y: Vertigo

C: Vertigo. Dizziness.

Y: Medications for some illness.

J: Dizziness covers it.

S: Certain antibiotics.

Y: Yes, combinations of strong antibiotics.

J: Why antibiotics?

Y: You feel drowsy.

S: Anti-histamine.

Y: Must take the non-drowsy one. For pilots, they use non-drowsy.

J: Anti-histamine is one of the main factors.

C: That means side effects of medications la. How about physical conditions?

Y: Disability, and the blind. One blind just fell into the pothole. He said cannot be seen, so deep. So ask me for help and people say eh, why got sound ‘Help! Help!’. Ya, people who can’t see the…

C: Sis Yen, you mentioned disabilities people, like...can you explain?

Y: On wheelchair. Because on wheelchair, some they don’t know how to use properly the wheelchair. They want to go up like a one inch or two inch curbs ah. They don’t know how to go up, they must step on the…at the back there are two rods, isn’t it? Near the wheel, they must step on the wheel, so the front heel get lifted up, then they go up. They don’t know, been struggling. Somebody from the front, will lift it up, so then there’s a danger of falling.

C: Anything else?

(Paused for 5 sec)

C: We proceed to the next question. Can you tell me some methods that you know in preventing falls that you practice in your daily life?

KH: The very important thing is, the moment you feel you are not steady, like not very balance, always recall, in your mind is, you squat down. I think that’s what my wife did.

Y: When you go dizzy, you squat down. So that your head is not higher…

KH: Then your injury will like, minimized or you are safe from injury.

J: (laughed) Like my mother ah, at her age, she cannot squat down. She squat down she cannot get up.

Y: Sit down. Never mind. Doesn’t matter.

KH: Never mind, at least you dint knock your head, and then you become unconscious, until no…If nobody around, you don’t know when you can get the help.

Y: When you standing, you head is higher level mah, then you fall, the impact is higher. On the floor, you fall also, not so great the impact.

KH: You will be surprise ah, if you keep on, bearing that in mind, when you do your meditation, there’s so many things you have to observe, mindful.

S: Practice balancing daily. Like I attend this PNF exercise, they teach you balancing. Standing in the figure of 1, with different, alternate your front and back leg, standing on one foot, so slowly you improved.

J: Balancing exercises. I see. Very good.

C: That’s a great point. Like any other exercises that you think can help?

CT: I think all forms of exercises, isn't it? It supposes to help our body. Isn’t it? You know.

C: Can you tell me more like what kind of exercises?

CT: I mean like Qi Gong…

J: Muscle strengthening ah. Muscle strengthening ah.

CT: Even for walk, you know?

J: Walk is weight bearing, so it’s also muscle strengthening.

Y: Especially core muscles strengthening. Like now I attend the gym ah, focusing on senior citizens, at Taipan there, you know. They focused more on senior citizens, and teach us on how to…exercise…just to develop that balance.

CT: Where? In Subang? Taipan?

Y: Taipan there.

CT: The brother’s centre there, is it?

Y: No. No. No. This is a different one, husband and wife, young couple.

C: Sis SH? Any methods that you know can prevent falls?

SH: Where’s my head ah..(laughed)

Y: Tai-chi.

C: Apart from exercises? (paused for 5 sec) Anyone else?

S: Does carrying a walking stick help…the old…on falling?

J: It helps to certain age only. It helps to certain age. Because the walking stick is not stable. So it’s always the better the walker.

CT: The frame.

Y: Or single frame, (J: (interrupted) Single is not so good.) with the leg one…

CT: With the four legs one.

Y: The four legged stick. Ah, okok.

CT: I think we should start off with the one la. (J: Of cause)Start off…Some even use the umbrella, you know, to look better (laughed), and then slowly la I think, we progress…progress into…

J: I don’t see shy if I use…

CT: I mean some people…

SH: Ya. Especially you are walking, and err you want to go…to the shop for makan, or to get something from the sundry shop, you have a step or two to climb. So if you are not very steady, so it is better to hold a stick, you know, that helps you, I will called it my third leg. (laughed) (J: of course, of course) I called it my third leg. (J: ya, it’s better than nothing you know) That will helps to take off some of the weight, you know.

Y: If you old also, it’s good to eyes check-up every year.

CT: Ya, the eyesight is important.

Y: Something glaucoma, you don’t know, is coming. Or…No denial la. If you think you got cataract, better go and see doctor. Like myself, everyday I postponed. Ya, ya, I must go and see…for my cataract.

CT: The eye. The ear also. The hearing…you know.

Y: Vertigo sometimes can due to the infection of the ear…I had a bad experience of vertigo. I was alone at home, I was standing in the kitchen and suddenly I bend down the bottom shelves to get something, you know. Wah, the whole house was spinning. (CT: The ceiling is like...spinning.) The whole house was spinning. I quickly just sat on the floor. And I have to sit for at least 15 minutes you know, before the turning goes off. That bad you know.

CT: That bad ah.

SH: I had a vertigo literally for three days. Yes, three days! My head was spinning.

Y: Then I moved to my room, and then lie down and took a nap. Then after that I went and see the doctor. Have to, have to. Because it lasts for few days.

CT: It happens to my niece, you know. She was in the 30s only what. The son was virtually crying away, you know, because really seeing his mother in that condition.

J: Temporary only ah?

CT: No. Hers was quite bad. And then err…called la the uncle and the auntie to come.

C: So, like getting regular checkup for the eyes and ears is important.

C: Sis S. Anything that you would like to add on?

S: I don’t think so, but I was…mentally I was questioning, why is it old people feel shy to carry walking stick. Many…

SH: 不好看啊 (cantonese)

S: 看(cantonese) is only the face, not your stick.

Y: Denial that you are old.

CT: They still want to feel…young and able.

SH: Self dignity.

Y: Self-image. Ego.

S: And I used to think it’s educational level. (J: correct) But no, you know. (J: Har?) I have one very intelligent lady. When she was old, but not that old, I used to say why is it this old people ah, when they have to carry a walking stick, they won’t. I said will you do that? She said ‘No if I need it, I will use it.’ Now she is 85, I think, she refuses to use it, you know, she told me that earlier, but at this age, she refuses to use it. I said ‘why?’.

Y: What did she said?

S: So, she can’t answer me.

SH: My sis in law also the same. Ask her to use, 不要 (cantonese), why, 不好看 (cantonese) , 我说你跌了就好看 (cantonese).

Y: Then she uses?

SH: When you fall down, then it looks better. You look better la when people see you dropping. (laughed)

KH: The consequences, they didn’t think about it. Think about the不好看(cantonese) first.

Y: The reality hasn’t sink in, about falling.

SH: The self-image ah.

S: They cannot accept that they have grown old.

SH: Yes. Yes. Yes.

CT: Mrs Cxx la, umbrella, she have been using that.

C: We will now focus on home modifications. Like any type of modifications that you can do at home so that you can prevent falls?

J: Oh, that one I can talk a lot.

C: Ok, Bro J first.

J: I think what helps very much was, very early on, when I got her here with me, I made a pole, right next to her bed, I can draw for you afterwards, I will draw for you. This is from the ceiling to the floor. This is just close to the bed. Close to bed, I put the portable commode. Anytime at night…This is the time that she will falls, when getting up and go to the commode, so she always has this hand pole. Always. That helps very much ah. I always here, and I trained her ‘Hold. Hold. Hold’. I will draw for you, before I leave.

C: Anything else?

CT: You have to get somebody to come in to fix it?

J: It was my idea and I get the person to do it. I am supervising. And hand holds la, watch where they are coming from the room, where they are holding the hands. Watch it, then you put the hand pole.

Y: The pole is wooden one?

J: No. No. It’s stainless steel. (Y: Woah…) From ceiling to the floor, you know. Very well placed.

S: Like the fireman’s type la.

Y: So people will think that she is staying upstairs. And then she wants to come down, zoom…

J: But you get them downstairs la. No upstairs. Definitely no.

CT: That’s why the downstairs room is very important.

Y: Must have a downstairs room.

J: I draw for you later.

SH: This is why I buy a single storey house.

CT: Even for us, we maintained. Some people knocked it down and turn into dining area. (J: Until they get old la) Then they said never mind. They will fix it back. My neighbour told me that.

C: Any other modifications that you can do at home?

CT: Bathroom, they must have the rail. (KH: the grab rail la) And then the toilet seat also. Those who have the squatting one, better go and change…into the sitting one. There are family…you know…

Y: They still have!

J: They have to change la, you know.

C: Anything else?

J: Railing is important.

SH: You know the Qxx ah, QCB, the other Qxx staying in Bangsar one. (Y: dentist, the son is a dentist)

CT: Ah…What’s his wife’s name already? (Y: Ah, why?)

SH: Her husband was a Parkinson. Parkinson…AF…

CT: Mrs Fxx. Mrs Fxx is Alzheirmer’s.

SH: Ya, that’s why, you see, their pathway to walk up to the house is quite far, you know. So from the house want to come out, the fellow is not steady, he got Parkinson. I said ‘Why don’t you put the railing there, so as they come out from the house they hold on…’

J: Correct.

Y: You meant the bathroom is outside?

SH: No. No. No. Walking up to the house. Up and down. From their house to the gate is quite a distance and is a bit sloppy, you know. Now, the wife…later, the wife has Alzheirmer’s. So, two of them.

KH: Oh, one Parkinson, one Alzheirmer’s.

SH: But they ended up in nursing home…old folks home.

CT: Many years already.

Y: Ya, another thing forget, the good lightning, especially at night. You must have a night lamp. And day time also, if there’s a staircase, they need a good lightning for those places

SH: You go to my house, I always put on light.

CT: I love to on it also. But my the other half was like, (Y: 浪费电(cantonese)ah) always like…why…ya,浪(cantonese)…I like it to be lighted, you know. If a place ah…very gloomy…isn’t it? But he just cannot agree.

Y: Like if you drawn the curtain, just open the curtain, open the door and all that, that’s brighter. Day light is better than the neon light la.

SH: Must always have a night light.

Y: At night, at least one lamp should be on, even the hall ah..People say浪费电(cantonese) The hall light also off. Don’t need to go to the kitchen. Off the kitchen light.

C: Sis S?

S: I think no need. Mainly two things ah. Hand rails and lightnings only.

Y: And no wet floor. Dry floor.

S: No she said home improvements. Now we are at home improvements.

Y: Oh. Oh. Sorry. Sorry.

C: Bro KH, anything that you would like to add on?

KH: Not I can think of now. (laughed)

C: The next question, can you provide me some simple methods that you know on how you can find out if you are at the risk of falls?

Y: Say again, sorry.

C: Can you provide me some simple methods that you know on how you can find out if you have risk of falls?

KH: Risk?

J: Mainly self-awareness, whether you are susceptible to fall. Self-awareness.

C: Self-awareness. Any…other simple methods?

KH: I think it is very difficult to prevent falls. It can happen…the…because…we have to accept that falling is like accident, and it is very difficult to prevent accident happen. But very important is that you recognize it, you have to keep yourself as, as, as healthy as possible, which means that even you fall, you are able to break the fall. That’s what I think.

C: It’s mainly self-awareness la…

CT: Like what you said, Mrs Lxx la. She knew that, you know, so she was squatted down, so the impact will not be that great.

KH: But, there’s this part that…If you start from when you are younger, you know, and you continuously be aware to actually do the necessary exercises, where your whole physique is more supple, I think it keeps diseases away also, it’s not only talking about falling and all that. And actually, that’s help. That’s help.

CT: Ya, I think that one is true, you know. Like my mum, you know. Somehow ah she got that kind of mentality ah, that she could do everything….she could do a lot, you know. She’s not that type that oh…you know, aiyo I cannot do this, I cannot do that. Even…that’s why sometimes I do give her credit. Even though she is illiterate ah…at the age of 30 something only she went for a license, driving license. And it’s oral you know…I meant the…the highway signs…on the first round. And the practical one, that one not an issue. Managed to get through, you know. So I really give her back. And then with that also ah, the signs all, because she is illiterate, but she could dare to drive one, even…anywhere.

J: Ya, you have to give them credit la…give her credit la…

CT: No. Actually, it’s in her, you know…it’s in her. It’s not easy to go and tell, you know…especially their age ah, you know. So somehow I feel…

J: Oh women are strong, man! Like my mother la.

SH: the important thing is that erm we know our limitation, so we have to be more careful, especially when we walk. You know, you trip on something, you make sure there is nothing in the way; You incline to slip, make sure you don’t step on something and slipped. And the floor is not wet, you know. And if you wear footwear, it is…

CT: It must be anti-slipped.

J: Fitting.

CT: The sole shouldn’t be flat.

SH: These are awareness, and we are cautious about that. I think that should prevent la…hopefully….

C: There’s actually some simple balance and strengthening tests that you can find out whether you are at risk of falls or not. So would you be interested to learn how to access yourself for risk of falls using those simple methods?

crowd: Of course.

CT: Yes, good idea.

Y: Can I just add…sorry, you were asking how to prevent ah. Some people when they renovate the house, they try to make it one level. So sometimes there are, you know, indoor steps, going from the kitchen to the hall or whatever, so some just try to level it, so there’s no steps. This is also a very good.

J: Yes, Yes, of course. That’s the main thing.

KH: But some houses very difficult to do that you know. (Y: when it is too high.) Some at the damansara jaya, those areas.

CT: Those split level one, those old days.

Y: So if you need buy a house for old people, don’t buy the split level one. SS2, there was one…

CT: Oh ya, ya, ya, three steps.

SH: A lot of people sell their big bungalow house, go and live in the apartment.

S: For security la.

SH: Security, whatever reasons la.

CT: That one also another set of problems. Stay in condo, you have condo problems.

KH: Yes. I know people who stay, they say, I will never stay in condo again.

Y: Maintenance.

(Bro Jack was done with his drawing) KH: Already drawn.

(Everyone is looking at the drawing) CT: Next to her bed…J: always the hand…Y: The commode also near there.

CT: Your mother is so lucky to have a son like you.

J: Karma la. It’s a Karma what. (laughed)

J: Yes, two of us only. Close by only what, I stay.

CT: Kelana only.

C: Ok, the next question. Research finds that many older people cannot get up from a fall. Tell me steps by steps what an older adult should do after a fall?

CT: After a fall ah?

S: Roll over. When you fall, you roll over to get up, with the help of your four limbs, you cannot get up like normal ah…(J: correct, correct.) you have to roll over.

J: But my mother ah, you know, two weeks ago, she…I don’t know what she is doing la, at 5.30, (KH: am?), came out her room…Ya, a.m. Fell. I don’t know, I was upstairs. The good thing is my air-cond was off on that day, 5a.m, I woke up for the gym. I hear the yelling, her voice calling for me, so of course, so, I so panic. I go down la. She sit there on the floor. ‘You help me get up. Help me get up.’ So, I help her get up. So, hold on to her, but no head trauma, that’s the main thing. Head trauma is the worst, you know, when you fall. So, she walks straight away. I still home, I still home.

C: That means she cannot get up by herself?

J: Cannot get up.

Y: Muscles are weak…

CT: Sorry. But sometimes also because they panic you know. So they sort of…you know…(KH: Paralysed ah.) Ah…they panic, and then they got fear…you know.

Y: Strength. No strength ah. 95.

SH: When I slipped and fell on the road the other day. Everybody panic. All wanting to help me, all wanting to carry me up. I said ‘wait, wait, wait’. Let me settle down first, balance myself. Everybody from everywhere want to lift me up, you know…1, 2, 3, they yearn me up.

Y: Could be a bad injury, dangerous.

S: You must not allowed them.

SH: I stepped on, you know the plastic sheet they use, (CT: Oh, yes.) like the…bags ah…the white one, the bigger one, they put the onions, potatoes and all that, (CT: the net one is it?) ah, those days we have the gunny sack, now they don’t have, they use this one. So I was walking down, chee cheong fan there, that side, going down the road, just with my friend, to go and eat chee cheong fan, she parked the car there, walked two steps, and I very nicely la see this thing on the floor, never mind, stepped on it, zupp, I went, flat on my back, literally flat on my back, remember one of my client, when I was doing the research, ‘I fell like a coconut tree’. (laughed) Then I was thinking, ya…that’s how I fell la, like a coconut tree. Slipped and just went down straight like that. But I was very lucky. My head no injury. My back no injury. But I landed on my left knee. The knee was twisted like that. That’s why I am limping now. I still got the pain. It was about 5 months ago only. And some more with my back surgery ah, the surgeon told me, make sure you don’t fall. Har, and then I said ‘die this time’, land on my back.

C: So at that particular moment, can you get up by yourself?

SH: I couldn’t. I couldn’t. As...from the lying down like that, flat on my back, try to get up, I have to sit down first. Then they want to yearn me up. I said wait, wait, wait, hold on. But I got bad knees, you see, I cannot bend my knees. I got a lot of problems la. So anyway, I need help, so I said wait, wait till I steady myself…Put my leg down, put my feet down. Ah…then ok.

CT: You find your balance la…so you know which one…Like for me, I cannot…

Y: People who wants to help you is the most dangerous. Because when I slipped from the massage bed ah, quite high you know, and she dint know I injured. She pulled my hand, on…pull my injured hand, you know, that could have caused the fracture to be worse.

KH: Aggravated. Become compound fracture.

Y: The surgeon also asked, eh, very nasty crack la, you know. She tried to…the masseur was so panic ah, she tried to lift me up from the floor, on my injured hand.

KH: Like this sort of things. I would, I will never step on. Even I walk anywhere, if there’s man hole, I don’t walk on the…I don’t trust this country, (CT: go round it ah.) I walked…I stepped on the edge where the frame is, the chances of the frame giving way is very rare, right? Then…

SH: But you can never tell. (laughed) You can never tell.

J: He is engineer. He is engineer.

KH: But I keep on training myself, so even I step one that gives way. I am able to adjust my body.

J: Don’t jump in shit man.

Y: Usually I…to be on the safe side, I put one leg on it first, to see whether it is steady or not, whether it will crack or not. Then it’s quite firm ah, then I put my next leg.

SH: Never test it. Don’t test it. Don’t try your luck.

KH: Very risky. Very risky.

C: Do you think that like learning how to get up steps by steps is very important to you?

(everyone): Yes

J: Definitely, like what she was saying, you know, you got to roll over and get on your fours…But then you cannot chit-chat to a 85 or 90 years old person, you know.

SH: The muscle is not strong enough to lift you up.

Y: There should be a bell or something…

J: For us, yes. I meant I fall from my bike, I still get up. They are all running to help me. But I am still standing.

Y: Another thing they should have is, always have a bell at the bedside, like hospital, they have a bell.

J: I worry she will makes too much noise. She will be ringing the whole night.

Y: Or whistle

J: I think a whistle. A whistle.

S: But you will never fall near your bed, you will be falling away from your bed. How you going to crawl…how you going to your bell.

Y: Have a whistle in your bathroom. One whistle in your bag.

CT: No, carry a whistle la.

Y: It’s not expensive.

J: There’s no point if no one is in the house.

Y: Handphone la.

CT: Handphone la I think.

J: She don’t know how to use handphone.

Y: Teach her how to…just press one button only, to call the son.

J: No la. I got my neighbour’s maid. She can always access. When I go away 3 hours, I will tell my neighbour’s maid, you come over, every hour.

Y: My mum was 80 something. So we give her a handphone and programmed it, three person to press. So you press one button, somebody will respond.

J: That’s good. That’s good. She knows what button to press.

S: Now you know, it’s so sad to grow old.

(laughed)

CT: At least we ah…I meant we have all the knowledge.

J: Now only I realize. The first noble truth, sufferings. Now only I know.

CT: Now only ah?

J: When I come to this age. Now. But earlier never.

Y: No. No. Buddha said there’s a way out of suffering.

Y: No. No. No. Buddha said it is not so fatalistic one. There’s a way out from the suffering. And he teaches us the way.

CT: Now we are learning the way out already.

S: But the way is long long way.

Y: Once you are from young ah…

S: To get out from the suffering that means you are going to Nirvana you know. You know how long the journey is…The journey is long.

Y: But the two causes…Your craving and attachment, that’s all. Attach to your health, attach to your youth. And then craving, you crave for, you know…nice food.

J: Ok. That’s Buddhism ah. Let her finish la…

C: As you all know, we are going to design a website for the elderly. Can you tell me what kind of information you would like to have in this website?

Y: Big font. Hehe.

C: Oh no. Information.

Y: Oh Oh sorry sorry.

CT: What you want to know from the website.

J: It’s what we have discussed so far la. Falls prevention. Number one is falls prevention.

J: Self-awareness of your own limitations.

Y: Awareness of your surroundings.

J: Of course very important is always the caregiver, whether he is there.

C: That means the caregiver education? Anything else? Apart from what we have discussed just now?

J: We have discussed most of the things here already.

C: Any extra information that you would like to know?

J: For them is strength training la.

C: (repeat) What type of strengthening exercises?

J: Strength training exercises.

J: That’s only so much a person can do. There’s a lot on the caregiver. A lot.

KH: Ultimately…

C: Sorry, Bro KH?

KH: I can foresee is, ultimately the caregiver will need to know more, to be able to handle the situation. (J: The maid should be train.) It’s a training.

SH: Elderly care for themselves. We are talking about self-care.

J: Self-care is until certain age only.

S: I think it is also important to teach old people how to grow old. Because the old people are so bloody demanding. Yes. And it’s very difficult on caregivers. So they ought to be taught how to grow old gracefully.

J: If you can la…

KH: It’s a very challenging part. I remember my mother got a stroke. My elder sister was looking after her. She will use this…the wooden leather. And she will knock the bed, the wooden board, every time she wants anything…You are talking about phone, at least phone not so…this one is knock knock knock…(CT: so is non-stop.) My sister as a caregiver, become mengamuk la…

Y: Very stressful.

Y: Burn out.

CT: I think the family members should co-operate la…take turn, otherwise…ya, really burn out.

J: I have to do it myself, because they are not here conveniently.

S: I think statistics showed that 1 sick person, you need 5 caregivers. That’s the ratio, you know…for you to be comfortable for the caregivers, you need 5 to look after 1. I think that have taken the statistics…

CT: Your second brother?

J: One in Melaka, one still in US.

CT: Three is it?

J: Because my mother choose to stay with me. They all got their own family la.

Y: You know, they must take turn, at least.

CT: Conditions most. (laughed)

SH: Always falls on one person.

J: That one who she is willing with.

C: The primary caregiver.

Y: Sometime they contribute to pay for a maid. They contribute.

J: I yet come to the stage, because I say let me do this, until I cannot take it.

Y: No, once you feel you cannot do it, you are burn out, it’s too late already, get part time and all that.

Y: No, maybe some part time maid.

J: I travel la. I travel.

Y: Then who takes care?

J: I get…there is a lady opposite my house, she is about 40, 40 something. And for her, she is not earning any money. So reversal…so she just come over…

CT: Oh…that…the few hours when you are not around?

J: Oh, when I travel, I will be away for 10 days, 2 weeks.

KH: It’s quite a long time.

CT: I think yes, caregiver needs to take a break.

J: Ya. Ya. We need a break.

Y: Or even now we are taking care of her, but do you get a part time maid to clean the house…(J: I have. I have. Just to clean the house…I can’t do it.) clean the toilet, or even do some simple cooking ah…

J: Cooking ah…I go and buy food la.

C: The following questions will ask you about the characteristics of the webpage. Your views on the website design, when implemented, will make the webpage more accessible to all older adults. So, what are the characteristics of the webpage that will attract you to use it?

Y: User-friendly.

CT: You know, we go there for info…or info for health…

SH: Don’t ask me. Don’t ask me, I am blind to all these.

J: Ya, ya, me too. I am not IT savy.

C: Sis CT, what you have mentioned just now?

CT: No, I meant…We normally go for info what. That’s why you google...you know.

J: Make sure when you google, it gets there, rather than going through so many sites.

C: Like once you click into the website, like what you expect to see.

J: Easy to get in.

C: Easy to get in.

KH: It should be more…user-friendly ah.

J: Then you are talking about our age group, not very IT savy…

SH: Elderly user friendly.

J: Ya, we are not the young people.

C: Any other characteristics that you want from the website?

J: I think this one, you probably have to discuss with your contemporaries, the ease of getting to your site.

S: The younger people.

Y: They want our suggestions, so that they can…

C: We have the guidelines, but we want to hear from you all.

CT: Oh, they already have the guidelines.

C: Like, how to make the website more senior friendly.

SH: Maybe you show us, then we can tell you. You asked us how, then sorry la…

C: Ok, so we…

S: Larger font for the old. Pictorial for the old. Because we cannot read all these…

CT: Ya, fine prints ah…

Y: The eyes get very tired, when there is too many prints…too many sentences, so maybe break in paragraph.

SH: More info, less words.

J: Graphics ah. Graphics.

Y: Cartoons…colours…

C: We are going to show you some examples of websites, ok? Then I will give you some papers, you just write it down what you like and dislike of that particular website. So we can have a reference on what type of website you are more favour of.

SH: I am website out. (laughed)

CT: Oh they have the laptop.

C: Okay. Sis, Bro, can we continue?

C: We have seen through all the three websites. So we will discuss it one by one. Ok? We will start off with the first website. What do you think about the first website?

CT: The print is a bit small.

J: The font size is a bit too tiny.

S: I think you need to make it more attractive.

CT: And the colour is quite dull.

S: Not eye catching. Make you want to go to sleep.

C: Anything else about the first website?

C: Font small, colour is a bit dull.

Y: And the heading should be a bit bold la. Because it is a bit light ah…the heading, especially on the left hand size. And also like the capital letter la. Like ‘Key facts’ ah…’Key’ there is a capital letter, but ‘facts’ didn’t have the capital letter. It will make it even more…eye catching.

C: Now proceed to the second website.

J: The font size is correct.

J: Ah…Too many adverts.

CT: The distraction, because of the advertisement.

S: I find…easy access, pretty user friendly…in alphabetical order. Erm…The briefs at the side, at the right hand side. It helps you to search, you know, when you turn to that page…

C: That mean the links beside there?

S: What do you called? I called it briefs. I don’t know. I don’t know computer language.

Jade: The content summary. The briefing. The content.

S: That’s help. Instead of going down the page, you know you are going to see all these in this page. And there was a picture to help with, it cuts down the border, picture always speak thousand words.

CT, KH: That’s the third one.

S: Oh, that’s the third. Sorry. Sorry.

C: Ok, for the second website, anything to add on?

S: No attractive logos. 全部没有(hokkien) attractive logos one. You are going to the website, which is like looking at the face you know, when you look at the face, so when you turn to the page the logo刚才(hokkien) 没有的(hokkien).

C: Okay. Anything else?

C: Okay, now we proceed to the third website. What do you think about the third website?

SH: Simple.

J: The font size is correct. And then there’s no distraction. And it was quite concise on the sub-section.

S: There was a picture and I think that was good.

KH: Most ideal. The template is the most ideal.

C: So everyone agrees that the third website is the most suitable?

S: All the lao kok kok (hokkien) would agreed. (laughed)

C: Just now you all have mentioned on the font size, if I have the option that, so that you can increase the font size based on your…your…

CT: I think…that will be…helpful.

J: That will be helpful la. Because not many people are very familiar with this keyboard thing.

C: What do you think about language option? Like the first website, you can change it into mandarin, bahasa…

CT: I wasn’t aware of that.

CT: I think that helps also, you know…

Y,J; Mandarin.

Y: Oh, we didn’t notice ah…

CT: Also, bm also.

J: I don’t know how they read. For me, I don’t know…the malays ah…don’t look for…

SH: The young can read for the old.

CT: And then those in the Chinese one…the mandarin one, nowadays the young one…a lot you know.

SH: Because they go for their primary level edu…Chinese ed.

J: I notice a lot of the MO in UH are all mandarin speaking. A lot man…

CT: A lot of the doctors are mandarin speaking.

Y: Chung Ling high school every year get a bout of the…medical school is on Chung Ling high school, Penang.

C: How about speech function?

Y: Ah ya, I want to mention that. For those who are cannot hear…

C: Anyone noticed that the second website, there’s actually a speech function.

Y: Oh, I dint notice.

C: When you click on it, you no need to read the text, they will read aloud for you.

Everyone: Oh, that’s good.

Y: Ya, I just want to mention that…sound…for the blind ah…

J: That should be good.

C: Everyone agrees that it will be necessary for the elderly?

Everyone: Yes, yes

SH: Ya, ya, for people like me la…Because I am computer blind. I can hear, I cannot see.

C: Okay, how about colour? What colour you all actually prefer?

CT: Something bright.

J: Ya, bright colour.

SH: what you called that, pastel colour, the white is so glaring ah…

CT: No, no. I think pastel will be quite dull what.

SH: Light pastel la, light…

Y: Light blue, light green…

S: You see, the first screen is pastel, light blue and all, but it looks so dull.

SH: No, you must choose some colour that is not dull la.

Y: Purple, light orange, red, pink…whatever.

SH: Light pink, maybe, I think, I don’t know, depends on how it comes out on the screen.

C: So sis Siew Hong and Sis Yen prefer some pastel colour. How about bro Jack, and bro Keat Hin?

J: I think, for eye catching, you need bright colour la.

KH: For me, it doesn’t really matter. It must be able to read…black and white, read…you can see and read, read probably. That’s all.

J: Ya, ya, that’s true.

C: Sis Choon Tham?

CT: I think something…a bit more on the brighter side…because…with age ah…

J: Ya, you need eye catching…

CT: Eye catching, isn’t it?

C: Sis Szu-ih?

S: I think you colour the main headings.

C: But the background I can remain as white or?

S: Off white, Black on off white

CT: Contrast la, you know…mixed la…

S: Red colour, or something bright. So…because sometimes we don’t read all, we only read the headlines.

Y: Ya, I do colour my headlines…usually when I give talks ah…

C: How about the use of mouse? Is it easy for you all to click?

S: Easy.

J: Mouse, ya, I think generally everyone can…everyone can…

CT: Have to use what.

Y: No, laptop, you use your finger you know, I don’t like the finger.

KH: the pad or the…I opt for the mouse, than the (CT: …than the finger ah…) finger ah…

CT: Because I think we are so used to PC ah. Desktop.

C: Do you find it difficult go to the button there and then scroll down or scroll up? Is it easy for you all?

J: No. The small thing.

Y: There is a page up, page down. I use page up, page down.

S: You give us a mouse.

Jade: Ah because of the scroll wheel, is it?

S: You just scroll up and down.

Jade: You were using the mouse to scroll up and down. You didn’t go to the border, is it?

S: Hmm. No problem.

J: Coordination is a bit problem. Coordination is a bit of problem.

J: Mouse is easier, as compared to the thing...

KH: But the younger people…

J: Oh, they are very fast, man.

KH: They don’t use the mouse one. My daughter also…

S: I notice we old people ah, when we use this ah, we type like that you know, you young people…right? I see that, they do that, and I cannot handle that, you know, I still have to use like that…

Y: I use two fingers.

S: 他们用(hokkien) thumbs one. Because they need the fingers to hold, they can’t use the fingers, they use the thumbs. And they are very fast with it. I tried, you know, I cannot do it.

Y: I will put it down. There is a stand.

C: How about the use of image, animation, video?

J: I don’t know.

C: For example like, we are going to like…teach you all how to prevent falls, like some exercises. Do you prefer it in words, or in picture, or in video?

CT: I think picture also…is a good idea.

Y: But sometimes the picture is still one, if it’s video…

I think video is better.

J: Video is always good.

S: Video is better.

C: So we prefer less words.

CT: More…action. Visual ah.

C: For the help and information section, like if you have any comments or questions, which way would you prefer to like keep in touch with the person in charge, like whether phone call, or email, or there will be a active chat room, so that whoever are online, you can chat with each other and discuss about something…whichever way you think is…

Y: Oh share experience and all that…I think mixed la.

J: I never really got into that. So I can’t comment.

S: You know, I am trying to visualize it, like in whatsapp, you just press on the microphone, and you speak straight away, and you get the reply immediate. Instead of having to type, for the other fellow to receive, and type back. I think that is much faster.

CT: Ya hor the voice one…I hardly use that, you know, I think got to use it more often. How ah? You just press ah…the whatsapp there…is it?

S: Like I communicate with Baduma, she doesn’t speak English. Pigeon hokkien. So I will use my pigeon hokkien and speak to her that way la…because I can’t speak cantonese, and I cannot type and she can’t type for me.

C: So you prefer the voice one?

S: So that’s more…

CT: 我不会用(hokkien) must really get down to…to type is also lazy.

S: You reply instantly. The main thing is instant reply. When you want an answer. When you are not sure, you want an answer, straight away the reply can come in. Whereas if you email and all, it takes time, you lose interest already.

J: Of course la. Of course.

KH: You are referring to emergency?

CT: Call la. I think what about…

S: Not emergency. This is a webpage. You read, you don’t understand.

J: Chat room, she’s talking about chat room.

S: Chat group, there will a group. Sometimes you are not lucky to have a group.

C: No. There will be an active chat room. Whoever online, they can just type something there, and then like maybe bro Jack, type something and ask a question, and then when sis Szu-ih online, and she read the message and she can just reply.

J: Alright, I think that’s good.

KH: For that…actually, you need to actually type, but for voice, is one to one, because when there’s too many, you are talking to whom…

J: Chat room is better la.

C: So like ah to keep in touch with my supervisors, they all, they maybe looking through the website. Like if you have any questions, like which way do you prefer they can reply to you? Email?

Y: But we are not students, so we are…we don’t need to communicate with supervisors, so is different situations, isn’t it?

C: Oh no, like there are some questions that you would like to ask the doctors, or the physiotherapists.

Y: Maybe for start, Q&A, will be helpful. Q&A, the common questions ah..common questions and the specialists will reply. And it’s on the webpage. So anything extra, then maybe some communication or…way that we can communicate, or email.

KH: I think short notices, and all that, whatsapp ok, but for longer, anything that takes longer than one page or what, then maybe go to the email.

J: Whatsapp lo.

KH: Whatsapp is going to jam up the whole page.

J: Oh no no no. I am not talking about this.

C: Ok, the next question, can you share with me your thoughts on the use of IT for falls information?

J: For fake news ah?

CT: No. For this…’falls’.

J: Oh, sorry, sorry.

CT: I think is very helpful, you know.

J: Ya, it is.

KH: It’s time. Otherwise we will be going to left behind.

C: Sis Yen?

Y: Ya, ya, especially the common Q&A. So when I….usually go for the common Q&A, and all my questions are answered. I don’t even need to read much already…if it is a good website la…like this…

C: Do you like ever consider about the credibility of the information that you obtained online?

Y: Well-known websites la, like the doctors website, web medicine…

S: But we don’t know, you know. When you open, you really don’t know, what is good, and what is fault.

Y: You just key in the good…

S: Ya you can be reading, you can be reading fake news and you think it’s real. Some with pictures and videos inclusive, you see with your own eyes, but they are still fake. How do you differentiate that? Do you all younger people know how to differentiate?

C: Actually, we will go for the evidence-based one, that means it’s based on research, we got the information.

S: And how do you find out?

C: Ah…We got our own way. But of course, our website will be all based on research, so it’s like trustworthy one.

Y: Lecturers would have given us normally ah…lecturers will give you all, the students ah…these are the websites to go into, isn’t it?

C: The final question, if this web-based falls prevention education program was developed, what are your opinions about its use among the older adults?

Y: Say again…say again….sorry, old people ah, takes time to register.

KH: It involves more people you know.

J: It depends on how IT savy is your group, you know. It comes up to that. Not many 80, 90 can go on to the…and start reading you know…No.

You can start from the younger generation, as they progress…(J: you are talking about 70, 60, 70…80 I don’t know, not my mother) as they progress to the older age.

C: Sis Szu-ih?

S: Can you read that…as she was request it.

Y: Because my mind was thinking of something. Sorry.

C: If this website was to develop, what is your opinion about its use among the older adults?

Y: If it’s specifically made for the older adults, there shouldn’t be any problem, but the worst thing is you make it for the general public, and you hope that elderly also can use it. Then…it’s not…useful.

C: Oh…It is specifically for the older.

Y: Then it should be fine. Bear in mind of all these…aware of…

C: Sis CT?

CT: I think it should be more for the…for them, and also the caregivers. But caregivers always…will be…I meant…normally will be younger lo isn’t it, you know, or healthier.

SH: The education for the caregivers can be younger, is ok what, at least they also know, how to prevent it, and how to watch out for these, especially when they are caring for the elders ah…

Y: So what you mean is the website is meant for everybody la, young and old. Maybe specific information…

CT: Ya, ya, should be.

SH: How you focus only on the old? Can you focus only on the old? How do you how old I am, how old she is, how old she is.

S: I think it should caters for everybody, all languages, (SH: The young people would also like to know.) Malaysia mainly, the Chinese…Mandarin, Bahasa and English.

Y: what I mean is the information is for everybody, like she said about…the font, can be slightly, erm more user-friendly for the elderly, that’s what I meant.

S: Towards the end you can say, common questions asked by seniors. And there will be questions, answers, the seniors will be interested. Because the young people will be interested in certain values. And the seniors are different.

CT: Knowledge purposes. They would like to know for knowledge purpose.

C: We have come to the end of the discussion. Do you have any questions?

Y: Can I add what I mentioned just now…about the house…

C: Yes, anything else that you all would like to add on?

Y: The hazards in the house, ya, like wires ah, normally people will tape it up isn’t it, like you go to the shopping mall, so maybe if cannot avoid, then maybe you tape it up or you put it behind the cupboard and all that. Another thing is fire hazards. Because elderly cannot run so fast as the young, so usually when there’s a fire, usually the elderly the one who died in the fire, so the fire escape route must be always very clear, maybe fire drill with the elderly. The backdoor keys and all that should be very near to the backdoor. Not somewhere (CT: Somewhere hidden.) you find, you know…Those who live upstairs also, must have a live door…err…grill ah, where you will not fixed the fire hazards ah…

J: Ah ya ya. This is the door which is never secured. Anytime when there’s a danger, this is the door you get out, so never been locked, I never lock the house.

Y: Ya, the grill upstairs ah, shouldn’t be fixed until you cannot escape. There must be a…

J: We are talking about very old…

SH: Those days already all fixed.

J: No upstairs ah. You don’t come upstairs ah. This is whole different issue ah upstairs…

C: Bro KH?

KH: Upstairs is only the window. When you talking the grilled window, must be open and go out…When you have elderly like…

J: Their orientation is all gone

KH: Even my wife ah, even when there is emergency, she will go and find the keys…

C: Sis SH? Anything that you would like to add on?

SH: (shake her head)

C: Sis S?

S: No, I think we have covered all.

C: So that’s conclude our discussion today. Thank you for participating, we appreciate your presence and your views will be a valuable asset to this study. So we got some food prepared. So help yourself before you leave.

C: Thank you so much.
